# Development of the European Veterinary Medicines Gaps and Needs Compass for Sheep and Goats Based on Online Survey and Expert Knowledge Elicitation

**DOI:** 10.3390/vetsci13030297

**Published:** 2026-03-21

**Authors:** Nikola Čudina, Marina Marić, Lauren Chambers, Margot Vidalinc, Anne Katrine Aagaard, Javier Balado, Petra Bratić, Martin Ganter, Allan Hägg Grønborg, Hasan Hüseyin Şenyüz, Antonio Spezzigu, Aikaterini Pazarakioti, Clare Phythian, Rianne van Helden, Panagiotis D. Katsoulos, Arturo Anadón, Laure Baduel, Flore Demay, Rens van Dobbenburgh, Shereene Williams, Janos Kovacs, Jobke van Hout, Frane Božić, Nancy De Briyne, Wiebke Jansen

**Affiliations:** 1 Faculty of Veterinary Medicine, University of Zagreb, Heinzelova 55, 10000 Zagreb, Croatia; ncudina@vef.unizg.hr (N.Č.); pbratic@vef.hr (P.B.); bozic@vef.unizg.hr (F.B.); 2 Federation of Veterinarians of Europe, Rue Victor Oudart 7, 1030 Brussels, Belgium; marina@fve.org (M.M.); nancy@fve.org (N.D.B.); 3 Kvægdyrlægerne Midt ApS, Klintevej 3B, 8653 Them, Denmark; ak@kvaegmidt.dk; 4 Diputación de Castellón, Ares del Maestre, 12165 Castellón, Spain; jbalado@hotmail.es; 5 Clinic for Swine and Small Ruminants, Forensic Medicine and Ambulatory Service, University of Veterinary Medicine Hannover, Foundation, Bischofsholer Damm 15, 30173 Hannover, Germany; martin.ganter@tiho-hannover.de; 6 Distriktsveterinärerna, Jordbruksverket, 551 82 Jönköping, Sweden; allan.hagg.gronborg@distriktsveterinarerna.se; 7 Department of Animal Nutrition and Nutritional Disease, Faculty of Veterinary Medicine, University of Necmettin Erbakan, 42310 Konya, Türkiye; hasansenyuzvet@yahoo.com; 8 Embryosardegna, Tecnologia, Riproduzione e Fertilità, 07034 Perfugas, Italy; antospezzigu@gmail.com; 9 Clinic of Farm Animals, Faculty of Health Sciences, School of Veterinary Medicine, Aristotle University of Thessaloniki, 54627 Thessaloniki, Greece; apazarakioti@gmail.com (A.P.); katsoulo@vet.auth.gr (P.D.K.); 10 AkoVet Ltd., Palmerston North 4440, New Zealand; info@akovet.org; 11 Ovivet Schapenpraktijk, 5476 VW Vorstenbosch, The Netherlands; riannevhelden@hotmail.com; 12 Department of Pharmacology and Toxicology, Faculty of Veterinary Medicine, Universidad Complutense de Madrid, 28040 Madrid, Spain; anadon@vet.ucm.es; 13 Agence Nationale de Sécurité Sanitaire de l’Alimentation, de l’Environnement et du Travail, 94701 Maisons-Alfort, France; laure.baduel@anses.fr (L.B.); flore.demay@anses.fr (F.D.); 14 Faculty of Veterinary Medicine, Universiteit Utrecht, Yalelaan 1, 3584 CL Utrecht, The Netherlands; rvdobbenburgh@gmail.com; 15 Brooke, Friars Bridge Court, 41-45 Blackfriars Road, London SE1 8NZ, UK; shereene.williams@thebrooke.org; 16 European Medicines Agency, Veterinary Medicines Division, Domenico Scarlattilaan 6, 1083 HS Amsterdam, The Netherlands; janos.kovacs@ema.europa.eu; 17 Royal GD, Arnsbergstraat 7, 7418 EZ Deventer, The Netherlands; j.v.hout@gddiergezondheid.nl

**Keywords:** European Union, goats, lack of availability, medicine shortages, sheep, small ruminants, unmet needs, veterinary medicinal products (VMP)

## Abstract

Limited availability of veterinary medicinal products (VMPs) for small ruminants is a long-standing challenge. This mixed-methods study provides the first systematic definition and assessment of (i) shortages, (ii) lack of availability and (iii) unmet needs for sheep and goats across Europe. Survey data from 96 European veterinarians in 13 European countries (the majority of whom were from Spain, Germany, France, and Greece), a market analysis of authorized and available VMPs via the EMA Union Products Database (UPD) and expert knowledge elicitation (EKE) by 23 specialists were combined. Antimicrobials (36.7%) and nonsteroidal anti-inflammatory drugs (NSAIDs) (19.9%) were identified as the VMP categories most commonly perceived as critically important. Although nearly 5000 VMPs are authorized for small ruminants at the EU level, UPD market research reveals that there is a reported market availability of 28.9% of ovine and 32.7% of caprine authorized VMPs. Validation by EKE confirmed lack of VMP authorization to be the leading root cause of the lack of availability of ovine (31%) and caprine (43%) VMPs at both the national and EU level. The European Veterinary Medicines Gaps and Needs Compass identifies four highest-priority medicine groups lacking in availability for sheep (anthelmintics and endectocides, bacterial and viral vaccines) and two for goats (anthelmintics and bacterial vaccines). Moreover, 13 highest-priority unmet needs were identified for sheep and 14 for goats across antibacterials, analgetics and anti-inflammatories, antiparasitics and vaccines. Potential actionable solutions advised through EKE include harmonized market access pathways and targeted development (especially for vaccines, NSAIDs, and antibiotic teat injectors) to secure animal health, welfare, and One Health objectives.

## Simple Summary

Sheep and goats often lack essential veterinary medicinal products (VMPs) across Europe as these are either not authorized, not marketed or not developed yet. This mixed-methods study involves surveying European veterinarians working with small ruminants, analysis of the EMA Union Product Database for VMPs, and expert-led prioritization of VMP needs gaps. For all reported problems, experts provided specific root causes and potential solutions. This combined approach identifies the following most frequently recorded VMP lack of availability: vaccines; pain relief medicines (analgesics); and specific antimicrobials against mastitis and dewormers (anthelmintics). Furthermore, only one third of authorized medicines for sheep and goats were found to be recorded as available in the EMA Union Product Database. Additionally, experts indicate that many required medicines critical for daily practice are not authorized in European countries, necessitating veterinarians to resort to “the cascade” to meet certain pharmaceutical requirements. The described European Veterinary Medicines Gaps and Needs Compass for sheep and goats may support and guide relevant stakeholders in increasing availability of authorized VMPs. In this way, current data and knowledge may be better understood and usefully translated into action.

## 1. Introduction

The One Health approach recognizes the co-dependency of human and animal health at the human–animal–plant–environment interface [1]. Treatment and management of diseases is impossible without access to appropriate medicinal products [2]. Lack of appropriate medicines is a common challenge both in human and veterinary medicine [3,4]. Reasons may vary geographically and temporally. Medicine gaps are influenced by: increasing complexity and globalization of supply chains, economic and market pressures, regulatory and logistical challenges, global events such as geopolitical instability, extreme weather events and pandemics [3]. Compared to human medicine, veterinary medicine faces additional challenges, such as interspecies differences in patients. Veterinary medicine holds a relatively small market share (around 4% of the total medicines market), with limited commercial incentives [5,6,7,8]. Yet animal health is a crucial component for the fulfillment of global sustainability policies. The reduction in animal disease burden, including zoonoses, as well as the threat of antimicrobial (AMR) and antiparasitic resistance, are prerequisites for public health, food security and sustainable livestock systems [9,10,11,12].

Numerous health challenges in animals lack proper control tools. Existing products fail to sufficiently address mounting challenges in veterinary medicine, including alterations in animal husbandry practices, consumer expectations, regulatory requirements and withdrawal periods [11,13,14,15].

The European Union (EU) proposed several measures in Regulation (EU) 2019/6 to facilitate access to suitable medications for veterinary professionals. This includes special authorizations for limited markets, such as less comprehensive data requirements in authorization of veterinary medicinal products (VMPs) for animal species other than cattle, sheep for meat production, pigs, chickens, dogs and cats. For these products, the benefit of availability is greater than the risk associated with reduced documentation being provided during the authorization procedure [16,17,18]. Important shortages of critical human medicines and their active pharmaceutical ingredients (APIs) were identified by the European Commission’s Health Emergency Preparedness and Response Authority (HERA) as a threat for public health, human health care systems and Europe’s security [19]. Investigation to describe medicine gaps and associated root causes is therefore necessary. Veterinary stakeholders such as the Federation of Veterinarians of Europe (FVE) have been working alongside the European Medicines Agency (EMA), the Coordination Group for Mutual Recognition and Decentralized Procedures for Veterinary Medicinal Products (CMDv), the Committee for Veterinary Medicinal Products (CVMP), the Heads of Medicines Agencies (HMA), and other key stakeholders [14,20] to address this issue.

The EMA and the HMA released the first “shortage” definition agreed within the European Economic Area (EEA) in 2019 to address shortages and enhance communication [21,22]. Improving the availability of medicines authorized in the EU is a key priority for the European medicines regulatory network, including EMA [23]. To tackle disruptions regarding supply of medicines and ensure their continued availability in the EU, the HMA-EMA Veterinary Strategic Focus Group, the EMA Medicine Shortages Single Point of Contact (SPOC) Working Party and the HMA Working Group of Communication Professionals (WGCP) are providing strategic and structural solutions. Increasing availability of VMPs is an objective within the EMAs 2028 network strategy [24].

In response, FVE has launched the “VetMed+ Coalition initiative” to develop European Veterinary Medicines Gaps and Needs Compasses, categorized by species and by country, building on existing resources, including: Cartography of Therapeutic Gaps in France [15]; the World Veterinary Association’s (WVA) Essential Veterinary Medicines List for food-producing animals [25]; the World Small Animal Veterinary Association’s (WSAVA) Essential Medicines List for Cats and Dogs [26]; the World Organization for Animal Health (WOAH) List of Antimicrobial Agents of Veterinary Importance [27] and the FVE FishMed+ Coalition for Fish Diseases Lacking Treatment, and the Gap Analysis Outcome [14]. Additionally, insights will be incorporated from recent reports released by the Spanish, French, Portuguese, and Irish medicines agencies, highlighting the lack of availability of antimicrobial medicinal products and alternatives [28]. These compasses aim to provide guidance to marketing authorization holders (MAHs) and competent authorities (CAs), encouraging collaboration to ensure access to priority VMPs across Europe [29].

Pharmaceutical companies focus their resources on animal species and clinical indications which are financially more viable [5,6,7]. Among others, small ruminants are greatly affected by this.

There are over 70 million sheep and goats in the EU [30]. EU countries with the highest density of sheep include Greece, Cyprus, Italy, Spain and The Netherlands. The highest number of goats is in Greece and Spain. Small ruminants (and buffalos) make up 4% of EUs milk production volume [31], with many protected designation of origin (PDO) products which significantly contribute to these countries’ economic potential and identity [32]. In addition, extensively kept small ruminants in remote rural areas contribute to landscape and biodiversity conservation [33]. Therefore, sheep and goats have been chosen as the initial species for the aforementioned VetMed+ Coalition activities under a specialized small ruminant (SRumi) subgroup SRumi VetMed+.

Possible reasons for the practical unavailability of VMPs were grouped into three categories: (i) shortages—temporarily unavailable authorized and marketed VMPs due to supply chain disruptions as defined in HMA Good Practice Guidance for Communication to the Public on Medicines’ Availability Issues [4,22,34]; (ii) lack of availability—authorized VMPs exist but are not available in the country due to a discontinuation of commercialization based on the decision of a MAH to not market the VMP as defined in the EU Implementation Guide (Vet EU IG) on veterinary medicines product data in the EMA UPD [35]; and (iii) unmet needs—a disease for which there exists no satisfactory method of diagnosis, prevention or treatment authorized (in the Union) or, such a method does exists, in relation to which the VMP concerned brings a meaningful advantage, as defined in the Commission notice: Guidance to Applicants—Veterinary Medicinal Products (C/2024/1443) [29]. The guidance is provided specifically for the purposes of applying Article 23 of the Regulation (EU) 2019/6 regarding applications for limited markets, e.g., no authorized vaccine exists in Europe for “Peste des petits ruminants” (PPR) infection, a highly contagious viral disease affecting sheep and goats (Table 1).

VMP shortages, lack of availability, and unmet therapeutic needs in small ruminants persisting despite regulations are not uncommon phenomena. Regardless of the existing marketing authorization (centralized or national) for a certain VMP, its availability in an individual EU Member State (MS) may not be realized. Likewise, authorized and marketed VMPs may be temporarily unobtainable, often going undetected by existing national shortage monitoring platforms. Article 114 of Regulation (EU) 2019/6 provides a mechanism for veterinarians in cases when there is no adequate medicine authorized or available for the terrestrial food-producing animal species concerned. It allows use of a medicinal product prepared extemporaneously or licensed in another animal species, (food-producing or non-food-producing) or humans, in exceptional circumstances to avoid unacceptable suffering. This is often referred to as “cascade use”. Such use is only justified under exceptional circumstances, to avoid unacceptable suffering and longer withdrawal periods, according to Article 115 of the Regulation [17]. This provides a flexible solution for irregular absence of VMPs but could also mask the real lack of availability.

The first aim of this survey study is to systematically identify and categorize the lack of availability and unmet needs of small ruminant VMPs across European countries. The second aim is to develop a descriptive structured European Veterinary Medicines Gaps and Needs Compass for small ruminants, with prioritization and identified root causes of problems. Furthermore, we aim to explore proposed alternatives and solutions to alleviate the recorded lack of availability, taking into account practical realities of veterinarians as well as the European regulatory and market landscape.

## 2. Materials and Methods

This study utilizes a mixed-method approach, with a sequential design to systematically identify, validate, and contextualize small ruminant VMP shortages, lack of availability and unmet needs across Europe. A triangulated approach was chosen, sequencing (i) quantitative online survey data, (ii) regulatory market analysis based on the official EMA UPD for market research and comparison of the official and survey-reported availability, and (iii) qualitative expert knowledge elicitation (EKE) from experts on small ruminants from different FVE member countries, with the aim of a result validation and identification of root causes, as well as potential remedies for identified problems.

### 2.1. Survey

An online survey for veterinarians working in the European Region was carried out. Quantitative data was gathered to identify general trends regarding the most frequent and impactful diseases, lack of availability of VMPs and unmet needs affecting small ruminants. The survey was developed by the FVE and qualified field experts, to ensure content reliability and validity of the survey, in accordance with the STROBE (Strengthening the Reporting of Observational Studies in Epidemiology) guideline for cross-sectional studies (Supplementary Materials, Table S1) [36] and the Checklist for Reporting Results of Internet E-Surveys (CHERRIES, Supplementary Materials, Table S2) [37]. Psychometric testing of the survey was not performed. Prior to distribution, formal proofreading and testing of the questionnaire was carried out by veterinarians, including native and non-native English speakers from different sectors and various European countries to identify potential interpretation difficulties. Any unclear questions were adjusted accordingly. E-mails with an open link to the questionnaire on Google Forms were sent to the 51 national veterinary associations of members of the FVE and allied stakeholders, such as the European Board for Veterinary Specialization (EBVS) and its relevant college of Small Ruminant Health Management (ECSRHM). The initial email and the two reminders on 22nd of May and 4th of June included a request to forward the survey to their respective individual members. The survey was available in English with an option for machine translation into all languages supported by Google translate. Minor translation inaccuracies cannot be entirely excluded. Participants were given appropriate project information, including content and purpose. They had the ability to review and change their answers before submission. Participation was voluntary and not remunerated. The online survey was accessible for 3 months, from 30 April 2025 until 21 July 2025.

In order to ensure a detailed insight into the real-life challenges veterinarians encounter with sheep and goat VMPs, the questions were open-ended and answers were standardized and harmonized by the method of lowest-level terms (LLT), referring to the most specific category in the hierarchical structure of the veterinary medical dictionary. The coding of verbatim answers from each country into appropriate LLTs was reviewed and approved by the experts from countries concerned (Supplementary Materials, Table S3). The LLT level provided the greatest specificity for coding, ensuring accuracy by representing the exact details of reported information. The survey was divided into five sections, with each consisting of a single open-ended question (Supplementary Materials, File S1).

#### Survey Data Handling and Statistical Analysis

Survey data was collected anonymously, with the choice to opt in to sharing of their email address. Any potential contact details or names mentioned by participants during the research were anonymized after transcription. Incomplete or duplicate responses based on time stamps were removed and the first entry was kept for analysis. Responses to open questions were standardized and categorized for harmonization purposes. Multiple answers were accepted to account for veterinarians working with both small ruminant species, resulting in varying group totals. After cleaning, data was tabulated, processed in Microsoft^®^ Excel, and organized according to criteria described in the Supplementary Table S3. Chi-square (χ2) tests were used to analyze differences between medicine categories reported by responders from clinical and non-clinical workplace roles. Fisher’s exact test was used for analysis of categories with an expected number of observations < 5. Calculations were performed in Statistica v14 (TIBCO Software Inc., Palo Alto, CA, USA, 2020). A *p*-value of ≤0.05 was considered significant.

### 2.2. Listing Authorized and Available VMPs for Small Ruminants in the EU Product Database

To validate the discrepancies between reported lack of availability/unmet needs and official market status, EMA UPD data was used as a reference. In the EMA UPD search filter, the species filters “sheep” and “goat” were used. Additionally, the EMA UPD contains products which are authorized exclusively for the specific species subcategory, such as small ruminants of certain age groups or production stages. These were mentioned separately in the final country-specific compasses. Only currently, at the point of analysis, authorized products were included in the data collection. Withdrawn or suspended authorizations were excluded from the analysis. The last update of the EMA UPD status in this study was carried out through January and February 2026 with the last accessed date being on 11 February 2026.

### 2.3. Expert Knowledge Elicitation

To ensure scientific validity, practical relevance, and stakeholder alignment of the results acquired with the online survey, a structured expert knowledge elicitation (EKE) was carried out to complement and validate the outcomes of the online survey. The EKE methodology followed recognized good-practice principles for elicitation, including iterative assessment, consensus-building procedures, and a selection of experts based on diversity of expertise and transparent selection processes. Inclusion criteria required ≥5 years of experience in small ruminant medicine, current practice in clinical, public service or academic roles. Experts from 38 European countries were invited, drawing on FVE members and professional networks, leading to 23 experts who consented and participated; namely with one national representative (Belgium, Croatia, Denmark, Netherlands, Spain, Sweden, Türkiye), two (Germany and Portugal) or three national representatives (France, Greece, Italy, and the United Kingdom). This is in accordance with internationally recognized frameworks, including the French national therapeutic gap cartographies, WVAs/The Brookes global essential medicines lists, and WOAH classifications of veterinary antimicrobials. These sources provided benchmark standards, facilitated cross-comparison, and strengthened the interpretability and relevance of the results. The SRumi VetMed+ EKE served to:

1. Review survey responses on priority veterinary medicines and unmet therapeutic needs for small ruminants through a structured rating instrument (Likert 5-point scales for importance/frequency). Ratings were submitted independently to minimize peer influence. Aggregated statistics and anonymized rationales were then circulated; a single virtual feedback session allowed clarification of item definitions but no real-time re-rating to avoid dominance effects.
2. Rank and prioritize the medicines identified on a scale of 1 (very urgent—to be solved in the next three years) to 5 (not urgent), based on criteria including: prevalence and impact of diseases, animal health and welfare relevance, evidence of efficacy, safety, cost-effectiveness and market availability.
3. Attribute, where possible, root causes for the lack of availability and unmet needs. The set of assignable categories for the root causes were adopted from the Cartography of Therapeutic Gaps in France [15] and Coordination and Harmonization of the Existing Systems against Shortages of Medicines—European Network Analysis report on root causes [4] (Table 2).
4. Reach consensus on a draft “Compass of Lacking Priority Medicines and Unmet Needs” by species. Pre-registered decision rules specified that only a maximum of five VMPs of the highest priority aligning with EMA UPD trends would be escalated to the compass; otherwise, items were noted for further study. Dispersion by country was tracked to identify context-specific divergence.

**Table 2:** List of root-cause categories for lack of availability of VMPs for small ruminants. The table shows an explanation of possible root causes which can be attributed to a problem reported in the survey. Root causes were assigned an appropriate tag in the brackets.

Root Cause (Tag)	Explanation
Distribution issues and shortages (Dist)	Includes quality issues, manufacturing or supply chain issues, unpredicted major events or natural disasters, distribution issues, and commercial issues.
Regulatory (Reg)	Existing limitations within the regulatory framework that either prohibit the intended use or render it unfeasible in practice, such as MRL restrictions (e.g., for dairy production), or withdrawal period issues (e.g., too long for lactating animals), or when “cascade use” is difficult or not possible (e.g., restricted access to human medicines).
Absence of suitable VMP form (Form)	Authorized VMPs and suitable API exist for a clinical indication, but unsuitable for the intended use (unsuitable dosage (including dose, interval and duration), galenic form, route of administration, or duration of use for a specific clinical indication)
Perceived insufficient efficacy or safety (Efs.) of use	Efficacy or safety perceived as insufficient
Unmet needs on national level (UnmetNat)	No authorized VMP for the clinical indication/species on national level
Unmet needs on EU level (UnmetEU)	No authorized VMP for the clinical indication/species on EU level

### 2.4. Limitations

The sequence was designed to move from broad, perception-based insight to objective data triangulation to expert validation and explanation. The needs and experienced lacks of medicine availability from veterinarians may be perceived on many different levels and the survey allowed for the identification of front-line perceptions of challenges, priorities and blind spots. Some veterinarians tend to focus on a complete therapeutic gap, while some highlight a lack of a specific API even though other therapeutic options exist. The EMA UPD market research as the field survey data provides the basis for identification of needed medicines to test, refine, or challenge these initial perception-based signals. The EKE contextualized, validated, or corrected the data based on domain expertise with a secondary EMA UPD validation. The FVE informed respondents of the survey about its concerns about a lack of availability of VMPs, which may result in animal health and welfare consequences. This has the potential to lead to contextual bias, as the survey relied on voluntary responses from practitioners.

The linguistic accessibility, supported by machine translation and wide geographical coverage of the survey helped secure a sufficient sample size. However, minor translation inaccuracies cannot be entirely excluded, and even well-translated questionnaires can be affected by cultural interpretation differences. The use of a snowball sampling strategy also made it difficult to determine the overall response rate or sampling error and limits the ability to generalize findings based solely on voluntary participation. Additionally, the absence of psychometric validation could allow for measurement bias.

As most responses originate from only four countries, some countries have a small number of respondents, and therefore the priorities for these countries as seen in the final compass may not be representative of the complete list of needs for the respective countries. The compass therefore lists the reported needs together with the evaluated priority status attributed to an expert from the same country. Moreover, most responses originated from only four countries. More than half of the responses came from Spain, while some EU Member States provided no responses at all. This uneven distribution affects the representativeness of the dataset and limits the extent to which results can be extrapolated to a European perspective. Because the availability of specific VMPs for sheep and goats varies widely across EU MS, this geographical imbalance may have influenced survey outcomes and introduced bias.

Similarly, country-specific priority rankings were based on expert input together with survey responses. In countries with few respondents, these priorities may reflect the views of a small group rather than a nationally representative consensus. In addition, the FVE had informed potential respondents about its concerns regarding VMP availability, which may have introduced contextual bias, as participants were already primed on the issue.

Another limitation was the challenge of clearly distinguishing between “lack of availability” and “unmet needs,” which may explain the observed overlap in responses to these two questions. Despite these limitations, the survey provides valuable insights into real-world experiences of VMP lack of availability across Europe.

## 3. Results

### 3.1. Part 1: Survey

#### 3.1.1. Demographic Data

The survey study resulted in 96 submitted responses from 13 countries, listed in descending order of responses received: Spain (n = 54/93, 58.1%), Germany (n = 13/93, 14.0%), France (n = 5/93, 5.4%), Greece (n = 4/93, 4.3%), Sweden (n = 3/93, 3.2%), Italy (n = 3/93, 3.2%), Croatia (n = 2/93, 2.1%), United Kingdom (n = 2/93, 2.1%), Netherlands (n = 2/93, 2.1%), Belgium (n = 2/93, 2.1%), Denmark (n = 1/93, 1.1%), Ireland (n = 1/93, 1.1%), and Türkiye (n = 1/93, 1.1%). Three survey responses that contained no information for any questions except the demographics were excluded from further analysis.

The largest portion of respondents reported having more than 25 years of experience (n = 34/93, 36.6%). The rest of reported ages were classified into the following categories: 6–15 years (n = 27/93, 29.0%), 16–25 years (n = 23/93, 24.7%), and ≤5 years (n = 9/93, 9.7%).

Workplace responses were categorized into clinical roles and non-clinical roles, with five subcategories. The majority of respondents reported working in a clinical setting (n = 78/93, 83.9%), of which 69% reported working in a practice specializing in small ruminants, and 31% in a mixed practice. Non-clinical workplaces included academia and research (n = 7/93, 7.5%), state veterinary authority (n = 5/93, 5.4%), and other (n = 3/93, 3.2%).

#### 3.1.2. Disease Burden/Most Important Indicators Requiring Treatment with a VMP

**Figure 1:**
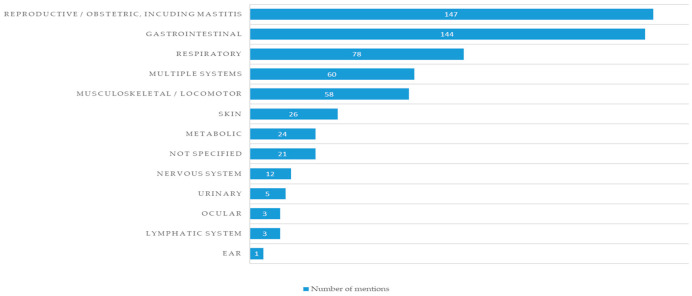
Disease burden categories for small ruminants. This figure shows the total number of diseases mentioned by survey respondents, grouped by the organ system they affect. The numbers shown represent the total distribution of answers between survey respondents.

Respondents were asked to write in their own words, based on their background experience, up to ten most common diseases/clinical indications affecting small ruminants in their respective countries. These answers were then categorized into accurate identification of the etiology, zoonotic potential, and organ systems. The total number of mentions of diseases affecting each organ system is shown in Figure 1. Specific organ systems most affected for small ruminants according to the respondents’ experience were: reproductive/obstetric diseases including mastitis (n = 147/582, 25.3% of total mentions, with 69 mentions of mastitis, and 67 mentions of abortion); gastrointestinal infections (n = 144/582, 24.7% of total mentions, 34 mentions of endoparasites, and 23 mentions of enterotoxaemia); respiratory infections (n = 78/582, 13.4% of total mentions, including 40 mentions of pneumonia, and 14 mentions pasteurellosis), and musculoskeletal/locomotor infections (n = 58/582, 9.9% of total mentions, with 28 mentions of foot-rot). When comparing the answers of respondents in clinical *vs*. non-clinical roles for mentions of a specific organ system, no statistically significant differences were observed.

#### 3.1.3. Critical Medicines

**Figure 2:**
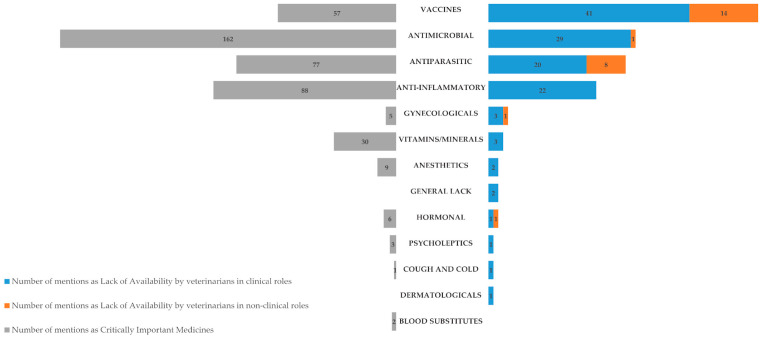
Reported critically important veterinary medicines (**left**) and lack of availability by category (**right**), in descending order.

The respondents listed in free text up to ten VMPs or VMP pharmaceutical categories they considered critically important, which they consider indispensable for their everyday practice. Figure 2 represents the number of mentions of each VMP pharmaceutical category reported as critically important by respondents. There were no statistically significant differences between clinical and non-clinical workplace role mentions of medicine categories. The most frequently mentioned medicine categories were: antimicrobials (n = 162/441, 36.7% of total mentions); anti-inflammatory, analgesic, and antipyretic drugs (n = 88/441, 19.9% of total mentions); antiparasitics, including endo- and ectoparasiticides and repellents (n = 77/441, 17.5% of total mentions) and vaccines (n = 57/441, 12.9% of total mentions).

Antimicrobial medicines for which the route of application was not specified were attributed to “Antimicrobials as a class”, and as such took up the majority (n = 149/162, 91.9%) of mentions within the antimicrobials category (Table 3).

Among the veterinary medicines mentioned in the “Anti-inflammatory, analgesic, and antipyretic” category, nonsteroidal anti-inflammatory drugs (NSAIDs) made up 53.4% (n = 47/88) of all responses. Medicine groups with frequent mentions in the category were corticosteroids (n = 18/88, 20.5%) and non-specified anti-inflammatory drugs (n = 14/88, 15.9%) (Table 4).

Mentions of anthelmintics and endectocides accounted for 74.6% (n = 50/67) of all mentions in the category of antiparasitics including endo- and ectoparasiticides and repellents (Table 5).

Vaccines were reported in multiple survey response categories, including etiology, clinical indication, affected organic system, or as a specific authorized VMP. They were categorized according to the modified ATCVet hierarchy (see Supplementary Materials Table S3). Most frequently mentioned vaccine groups were bacterial vaccines, with 76.5% (n = 39/51) of all mentions within the category of vaccines (Table 6).

Commonly mentioned vitamins and minerals were mostly different combinations of calcium, selenium and vitamins B and E, either as multivitamin preparations (n = 3/20, 15%), plain vitamins (n = 2/20, 10.0%), vitamin and mineral combinations (n = 4/20, 20%), and plain minerals (n = 5/20, 25.0%). Other specific categories mentioned were vitamin B complex (n = 3/20, 15.0%), vitamins for intramammary use (n = 1/20, 5.0%), and calcium in combination with other medicines (n = 2/20, 10.0%).

#### 3.1.4. Lack of Availability

Most respondents reported a lack of availability for both small ruminant species (n = 136/163, 83.4%), with some reporting only a lack for goats (n = 21/163, 12.9%) or sheep (n = 6/163, 3.7%).

Respondents provided further information about the perceived lack of availability of VMPs for small ruminants in their respective countries, such as detailing the specific products and species affected. A similar categorization of medicine subcategories was used, as in Section 3.1.3 (Figure 2). Vaccines were the most frequently mentioned category for both respondent groups (Figure 2).

Veterinarians in clinical roles mentioned significantly more frequently a lack of availability for all of the most frequently mentioned medicine categories except antiparasitic medicines (Supplementary Materials, Table S4).

#### 3.1.5. Unmet Needs

Respondents mentioned unmet needs in one of two ways: either diseases for which there is no authorized treatment or insufficient options within medicine classes or for affected species. Reported unmet needs largely align with the reported lack of availability. Similarly, the most commonly reported categories for pharmaceutical category unmet needs were antimicrobials (n = 38/144, 26.0%), anti-inflammatory medicines (n = 33/144, 22.9%) and vaccines (n = 28/144, 19.4%). Other categories of pharmaceuticals reported as unmet needs were: general VMP gaps and needs for small ruminants (n = 21/144, 14.6%); antiparasitic medicines (n = 15/144, 10.4%); alimentary tract and metabolic products (n = 3/144, 2.1%); anesthetics (n = 2/144, 1.4%); gynecological drugs (n = 2/144, 1.4%); and dermatological drugs (n = 2/144, 1.4%). No statistically significant differences in categories of medicines mentioned were observed between respondents in clinical vs. non-clinical workplaces.

Mastitis (n = 11/87, 12.6%), respiratory infections (n = 9/87, 10.3%), pasteurellosis (n = 7/87, 8.0%), and clostridiosis (n = 6/87, 6.9%) were the most commonly reported unmet needs by disease.

### 3.2. Part 2: Market Availability Analysis According to the Union Product Database

The EMA UPD search resulted in n = 4895 results for VMPs authorized for sheep in the EU, and n = 2151 authorized VMPs for goats. When using both “sheep” and “goat” filters, the database showed n = 5054 results due to an overlap with VMPs authorized for both species, of which n = 1450 VMPs were also authorized and available on the market for both species. Hence, around a third of all products authorized for sheep and goats were also recorded as available in the EMA UPD (n = 1415/4895, 28.9% for sheep; n = 703/2151, 32.7% for goats). Out of 207 unique LLTs mentioned in the survey, the EMA UPD search indicated an unmet need on the EU level (no authorized and available VMPs) for 65 ovine VMP needs (n = 65/207, 31.4%), and 88 caprine VMP needs (n = 88/207, 42.5%). Out of these, 60 unmet needs (n = 60/207, 29.0%) exist for both species. According to the EMA UPD, in all countries covered by the survey, unmet needs account for more than 50% of the reported LLTs, except for Spain, where 33.8% of respondents mentioned LLTs for sheep (n = 24/71) are unmet needs.

### 3.3. Part 3: Expert Knowledge Elicitation

#### 3.3.1. Survey Results Validation by the Expert Group

During the first expert knowledge elicitation (EKE), the experts were presented with the results of the survey. Firstly, the experts expressed their opinion on whether implementation of Regulation (EU) 2019/6 resulted in an expected increase in medicine availability for small ruminants. The experts agreed that, so far, the availability stayed the same or decreased (n = 6/8, 75%). No experts reported an increase in availability for either of the two species. One third of the experts expressed that the problem of lack of availability is equally affecting both species (n = 3/8, 37.5%). The experts unanimously agreed that the survey listed the most prevalent diseases and unmet needs affecting small ruminants and appropriately allocated the perceived frequency of the lack of availability per each medicine category. Emphasis was placed on mastitis treatment within the antimicrobial medicine category, as it had the highest reported prevalence for antimicrobial unmet needs within the survey (n = 11/87, 12.6%). Also, hormones and gynecological issues were discussed as a whole, since reported availability lacks and unmet needs were limited to oxytocics and sympathomimetics. The experts confirmed that all mentioned medicine categories suffered from a lack of availability, but also highlighted the lack of availability happening on a monthly or weekly basis for antimicrobials for mastitis (n = 6/8, 75%), vaccines (n = 4/8, 50%), and antiparasitics (n = 3/8, 37.5%).

As four categories of medicines stood out the most in all questions, expert knowledge was further elicited for anti-inflammatory medicines, analgesic medicines, and antipyretic medicines; antimicrobial medicines; antiparasitics, including endo- and ectoparasiticides and repellents; and vaccines. All but one expert expressed NSAIDs were very important in the treatment of small ruminants, but noted that this was the VMP group most often unavailable (Supplementary Materials, Figure S1).

#### 3.3.2. Root Cause and Solution Attribution

Upon completing the survey analysis and the EMA UPD reported the availability comparison for each country, experts assessed the priority levels, root causes and solutions for the reported problems in country-specific tables, with the list of medicines which were reported as unavailable by the survey respondents by VMP categories (Table 7 and Table 8). In addition to the provided details, the experts highlighted country-specific intricacies and concerns. Lower priority (priority levels 2, 3, and 4) gaps and needs for all countries are shown in the Supplementary Materials (Supplementary Materials, Table S5).

To address gaps in the survey-reported medicine categories, experts supplemented unspecified or lacking categories by adding specific medicines they identified as deficient within their national contexts. With this inclusion, the final LLT list counts 260 LLTs in total. Out of these 260 LLTs, 18.9% (n = 48/260) and 19.6% (n = 51/260) were ranked for at least one country as “very urgent, issue must be solved within next 3 years” (priority 1) for sheep and goats, respectively. Also, 34.2% (n = 88/260) of LLTs were ranked at least for 1 country as “urgent” (priority 2), 19.2% (n = 50/260) as “needed soon” (priority 3), 35% (n = 91/260) as “can be solved by the “cascade use” (priority 4), and 42.3% (n = 110/260) as “not a priority, authorized medicine exists for the species” (priority 5). The vaccines category was proportionally ranked as a top priority (n = 21/61; 34.4% for sheep and n = 24/61; 39.3% for goats). Overall, from the 260 LLTs, 136 referred to specific APIs and the most common attributed root cause for those VMPs mentioned in the survey and EKE with specified API based on the EMA UPD data was “Unmet need on EU level” (UnmetEU) (n = 60/136, 44.1%, for sheep and n = 88/136, 64.7% for goats). The category with the highest share of this root cause is anti-inflammatory medicines (n = 13/13; 100% for sheep and n = 10/13; 76.9% for goats). “Cascade use” was the most frequently offered remedy for the reported problems, mentioned 53/260 (20.4%) times as a solution for sheep and 59/260 (22.7%) for goats.

## 4. Discussion

To the best of our knowledge, this is the first survey-based study which aims to systematically identify and analyze small ruminant VMPs gaps and needs on the European level. Although root causes for medicine unavailability may often be similar worldwide, epidemiological, economic, and sociological specifics of individual countries result in significantly different needs. Since the majority of survey participants reported working in a small ruminant specialized practice, the survey results appropriately reflect the target population. The survey study aimed to gather real-world data on the problems which veterinary professionals regularly encounter when obtaining required medicines. This survey study provides the first assessment of unmet needs and availability issues in VMPs for small ruminants in European countries by implementing survey data across 13 countries complemented with EKE.

### 4.1. Market Structure Systematically Disadvantages Limited Markets

This survey demonstrates that significant and recurrent lacks occur in the availability of essential VMPs for small ruminants across Europe, despite the large number of EU-authorized products. A significant outcome of this research is the discrepancy between authorized VMPs and VMPs available on the market, as identified through the EMA UPD analysis. As shown in this survey-based study, a low marketing rate of authorized VMPs was reported as a frequent and ongoing problem in veterinary medicine [38]. Limited market species, including small ruminants, represent a low-volume, high-complexity market characterized by fragmented production systems, regional disease heterogeneity and diverse production purposes and practices. Therefore, authorization alone does not guarantee availability, especially in limited market species where the market is not economically rational for MAH, particularly where development costs are high and demand is uncertain [5,6,7]. The limited availability of VMPs for small ruminants reflects the structural constraints associated with limited market demand, higher costs for development, specialized pharmacokinetic/pharmacodynamic (PK/PD) requirements [6,8] and necessary in-depth knowledge of the pathophysiological peculiarities of small ruminants.

### 4.2. Regulatory Incentives Are Made but Remain Insufficient So Far

The root cause analysis and EKE in this survey-based study reveal that the underlying drivers are multifactorial, including distribution and manufacturing constraints, withdrawal period regulations, commercial discontinuations, and genuine unmet needs where no authorized VMPs exists. While regulatory facilitation aims to lower MAH burden, MAHs retain discretion for downstream decisions on commercialization, distribution, and national market entry. The experts agree with the broad consensus that, to date, the full impact of Regulation (EU) 2019/6 on its overarching goal of increasing medicine availability has not yet been demonstrated. However, in the RFSA cartography of veterinary medicine gaps, it was noted that the European Regulation had a positive impact on some milk withdrawal periods recommended in the case of “cascade use”, notably for NSAIDs and ectoparasiticides against flies [15]. Despite the incentives for limited markets introduced by Regulation (EU) 2019/6, such as simplified data requirements and special authorizations [17,18], the lacks of availability and unmet needs described suggest that these tools have not yet been translated into measurable improvements in on-the-ground access for veterinarians.

Prioritizing cross-border purchase facilitation, promoting the marketing of authorized products and addressing unmet needs are essential steps for improving animal health and welfare, supporting sustainable production, and advancing EU and “One Health” objectives.

### 4.3. VMPs for Small Ruminants Fulfill Public-Good Functions for Diseases Prevention and Control

While there is no universal list of common small ruminant diseases in Europe, the WOAH Priority Animal Diseases Sheets listed 30 zoonotic and non-zoonotic diseases deemed as global priority diseases in farm animals, offering a useful framework for identifying diseases of high relevance to small ruminants that may impact treatment prioritization [39]. Out of the 77 most important diseases mentioned in our survey study, eleven are listed in the WOAH sheet mentioned. These include sheep pox, bluetongue disease, contagious caprine pleuropneumonia, Q fever, tuberculosis, clostridiosis (black leg), dermatophilosis, fasciolosis, scabies, pasteurellosis, and intestinal nematodes. Additionally, although anthrax was not mentioned as one of the most important diseases in everyday small ruminant treatment, the vaccine for its prevention was mentioned as unavailable in Spain. The diseases reported by the respondents reflect the most common health issues of small ruminants. Reproductive and obstetric disorders, gastrointestinal parasitosis and respiratory diseases are considered important causes of economic loss and welfare impairment. The prominence of mastitis, abortion, endoparasitism, and pneumonia is in line with previous evidence that these conditions are the most frequent cause for pharmaceutical interventions [15,25,28]. Therefore, a critical but underappreciated dimension of VMP availability is that many essential medicines for small ruminants (particularly vaccines, antiparasitic, and antimicrobials against zoonotic disease), serve public-good functions. Without explicit policy mechanisms that recognize these broader benefits, commercial logic will continue to deprioritize “minor category species”, regardless of regulatory facilitation.

Regardless of their availability, it is vital to ascertain which medicines are critically important for everyday work in individual countries. This survey shows that anti-microbials, anti-inflammatory medicines, and antiparasitics were medicine categories predominantly recognized as critically important by European small ruminants’ veterinarians, from both clinical and non-clinical professional backgrounds. Both clinicians and non-clinicians identified vaccines as the most important and as most frequently lacking in availability. Clinicians mentioned significantly more often a lack of availability for all VMP categories except antiparasitics. This may be attributed to the focus of non-clinical roles on the prevention and management of infectious diseases, as opposed to the integrative approach in clinical settings, due to which veterinarians in clinical roles have field experience of the lack of various medicines (e.g., anti-inflammatory) and its direct impact. This highlights the importance of including clinically active professionals in activities such as the creation of country-specific lists of essential medicines. Comparing the results of this survey study question to the Essential Veterinary Medicines List for food-producing animals by the WVA and The Brooke [25], it is clear that the listed medicine groups overlap with the current needs of European small ruminants’ veterinarians to a great extent. However, within specific medicine categories, important differences are present.

#### 4.3.1. Range of Antibiotic Options and Ovine and Caprine Teat Injections Were Priority for Antimicrobials

Since the top priority diseases in small ruminants were mostly of infectious etiology, both the survey respondents and the EKE identified antimicrobial, anti-inflammatory and antiparasitic as the most lacking medicine categories. This is in accordance with findings from the French Cartography of Therapeutic Gaps [15] and the CHESSMEN analysis of root causes of shortage [4].

Route-specific intramammary antibiotics and clinically indicated antibiotics for mastitis, respiratory diseases, and gastrointestinal diseases accounted for the most frequent antimicrobial shortages mentioned in the survey. The WOAH List of Antimicrobial Agents of Veterinary importance categorized antimicrobial medicines used in the treatment of food-producing animals into Veterinary Critically Important Antimicrobial Agents (VCIA), Veterinary Highly Important Antimicrobial Agents (VHIA), and Veterinary Important Antimicrobial Agents (VIA) [27]. In our survey study, 68.7% (n = 22/32) of mentioned critically important antimicrobial medicines can be found in the VCIA category by WOAH, and 6.2% (n = 2/32). in the VHIA category. Out of the 32 antimicrobial medicines, 20 of them are also considered core medicines by the WVA and the Brooke list for sheep, and 18 for goats. Prudent and responsible use of antimicrobial medicines is of significant importance, as it can reduce the risk of bacterial resistance development. The need for such prudent and responsible use is especially highlighted in the use of antimicrobial agents that are used to treat both people and animals and are the “last line” of treatment for critical infections in people. The EMA Antimicrobial Advice Ad Hoc Expert Group (AMEG) created the Categorization of antibiotics for use in animals in which they categorized antimicrobial agents into four categories: category A (Avoid), category B (Restrict), category C (Caution), and category D (Prudence) [40]. More than a third of the antimicrobial agents reported as important in this survey-based study are found in category D of the EMA-AMEG categorization. This is a vital finding, since the use of these medicines is encouraged as “first line” treatment whenever possible. This indicates that in some situations certain policy measures and regulatory restrictions may contribute to the perceived lack of availability. However, more than half of mentioned antimicrobial medicines are AMEG C, which should be considered only when there are no antibiotics in the D category. All three antimicrobial groups from the AMEG B category were also mentioned, including colistin, ceftiofur, enrofloxacin, norfloxacin, and macrolides in general. Even though the B category of antimicrobial agents should only be used if no alternatives are viable from the previous categories and should be used based on antimicrobial susceptibility testing (AST), the need for these antibiotics for certain treatments of small ruminants is evident.

#### 4.3.2. Insufficient Therapeutic Options for Anti-Inflammatories, Antipyretics and Analgesics

Critically important anti-inflammatories, analgesics, and antipyretics were most frequently reported as unmet needs, which may be due to virtually non-existent therapeutic choices for small ruminants. One of the most important shortages and unmet needs is that of NSAIDs, which are essential for pain management and inflammation control, but also play a critical role in ensuring animal welfare. Specific APIs that are deemed critically important in our survey include metamizole, ketoprofen, flunixin, meloxicam, and dexamethasone. All mentioned APIs are also listed in the WVA/Brooke essential medicines list for small ruminants, with the exception of metamizole (also known as dipyrone), which was mentioned by only one respondent. Specifically, anti-inflammatories were reported as critical on the basis of their route of administration, with no regard to the medicine class. The routes mentioned include anti-inflammatories for ocular, topical, and intramammary use. Such route-based medicines are not mentioned in the WVA/Brooke essential medicines list. The list also mentions prednisolone as a core medicine, which was not mentioned as critical in our survey, but rather later on as an availability issue. Moreover, appropriate use of anti-inflammatories results in increased production and improved clinical outcomes [41,42]. Therefore, this unmet need represents a significant animal welfare concern.

#### 4.3.3. Availability of Critically Important Antiparasitics

Reported critically important medicines and WVA/Brooke essential medicines list overlap to a lesser extent when it comes to antiparasitics. At the therapeutic level, both lists cover anthelmintics, endectocides, and anticoccidials. However, the WVA/Brooke list does not cover ectoparasiticides, whereas survey respondents mentioned ectoparasiticides on multiple occasions, with a focus on pyrethroids [43]. On the other hand, the WVA/Brooke essentials list highlights the quinolone coccidiostat decoquinate, as well as the polyether ionophores lasalocid, and manduramicin, as essential anticoccidials, while our survey-based study shows that veterinarians considered halofuginone, toltrazuril, and diclazuril as important anticoccidials. Decoquinate, however, was mentioned later on in our survey as a medicine with a lack of availability. Notable discrepancies are seen in the benzimidazoles category where fenbendazole and flubendazole were not mentioned by the survey respondents as critically important, while triclabendazole was mentioned in combination with levamisole. Albendazole was mentioned in both lists. Shortages of antiparasitics, especially macrocyclic lactones and benzimidazoles, are of particular concern given the widespread anthelmintic resistance in Europe [44]. These shortages undermine the ability of veterinarians to implement evidence-based parasite control and increase reliance on the “cascade use”, which has been criticized as being suboptimal in terms of policy, pharmacovigilance, and animal welfare [45].

#### 4.3.4. Vaccines: Increased Availability Needed to Address Identified Gaps

The survey respondents stated the importance of vaccines for *Mycoplasma agalactiae*, *Coxiella burnetii*, contagious agalactia (*Mycoplasma* spp.), and *Chlamydia* spp. Conversely, these were not covered by the WVA/Brooke essential medicines list. Since survey respondents answered questions in a free-answer form, many of the reported critical vaccines were expressed as general vaccines for certain diseases without reported etiology. For such vaccines, it is not possible to verify if they are listed in the essentials list. These diseases include abortions, reproductive tract infections, mastitis, respiratory infections, and pneumonia. The WVA/Brooke list mentions vaccines against *Haemonchus contortus* and rabies as essential for both sheep and goats, “*Peste des Petits Ruminants*” (PPR) and goat pox in goats, and sheep pox in sheep, while no survey respondents mentioned these medicines as critical in their work. However, these vaccines were later identified as a priority by the EKE. Multiple respondents mentioned the importance of polyvalent combination vaccines against bluetongue virus serotypes which was also specifically noted in the essential list. Many of the respondents’ answers included specific brands of authorized vaccines for immunization against multiple species of microorganisms within the same genus. These vaccine products provided coverage for multiple diseases mentioned in the WVA/Brooke list, such as *Pasteurella* spp. and *Mycoplasma* spp. Whether these polyvalent vaccines were not considered appropriate for individual diseases was not stated. Vaccination in small ruminants is carried out at the flock level rather than individually. Major vaccinations include those against clostridial diseases, enterotoxaemia, infectious necrotic hepatitis, orf (contagious ecthyma), brucellosis, anthrax, agalactia, contagious caprine pleuropneumonia, *Salmonella* Abortusovis, *Campylobacter fetus*, pseudotuberculosis, foot-and-mouth disease, PPR, sheep and goat pox, and bluetongue [25]. In contrast, outside the EU, vaccines are available in Türkiye for brucellosis, campylobacteriosis, bluetongue, and PPR, whereas vaccines against chlamydiosis, salmonellosis, and toxoplasmosis are not available [46,47]. Vaccine shortages represent an important gap. Vaccines are classified among the most frequently unavailable VMPs, especially vaccines against bacterial diseases such as clostridial diseases, pasteurellosis, foot rot, and mastitis. This is alarming given that preventive vaccination is an important part of herd health programs and essential for reducing antimicrobial use—an EU regulatory priority emphasized in the Pharmaceutical Strategy for Europe [48] and WOAH stewardship frameworks [27]. Thus, limited vaccine availability may limit the contribution of disease prevention strategies to EU antimicrobial resistance objectives.

### 4.4. Using VMPs “Off-Label”: Inherent Constraints

The mentioned widespread reliance on “cascade use” (Articles 114–115, Regulation (EU) 2019/6) (i.e., use of medicinal products outside the terms of the marketing authorization) was often presented as a pragmatic solution for lack of availability. This is not consistent with the legal framework which refers to use under specific, exceptional conditions to avoid unacceptable animal suffering [17]. In contrast, the exceptional prescription of VMPs for deviation from the authorized dosage or route of administration (also known as “off-label use”) is defined as “the use of a veterinary medicinal product that is not in accordance with the summary product characteristics (SPC)”, which does not have the same meaning as “cascade use”, according to Regulation (EU) 2019/6 [49]. Exceptional prescribing constitutes a professional act inherent to the practice of veterinary medicine. In its application, veterinarians must act in accordance with their Code of Ethics and their academic and scientific knowledge, basing their practice and clinical judgment (“*Lex Artis* ad Hoc”) on the available scientific evidence. Furthermore, they may adapt the SPC of the veterinary and human medicinal products to the specific needs of their patients, while ensuring at all times respect for animal health and welfare, public health, and the protection of the environment. The EKE highlights that challenges of the “cascade use” (mentioned n = 53/260 (20.4%) times as a solution for sheep and n = 59/260 (22.7%) for goats in the Compass of all LLTs) relies on the transfer of legal and clinical responsibility to the veterinarian in charge, inconsistently applied across EU Member States. However, “cascade use” seems often to compensate for scarcity, allowing veterinarians to function without addressing root causes of gaps in availability. Frequent use of the “cascade” in small ruminants is therefore evidence of a systemic lack of availability. These findings reinforce the concerns raised by European regulatory bodies regarding persistent shortages and limited availability of certain veterinary medicines, especially those deemed important [3,15,21,28,35,48]. The major limitation of this survey-based study is the geographical imbalance of the respondents—particularly the predominance of responses from Spain and Germany—which may have influenced perceived priority gaps. This overrepresentation may be attributed to the apparent urgency for a solution regarding medicines availability on a national level. Such urgency may highly motivate veterinary practitioners to engage with activities such as this survey. However, the convergence with expert opinion suggests that the identified issues are broadly shared across Europe.

### 4.5. Increase Sheep and Goat VMP Market Availability: Multisectoral Approaches and Pathways Needed

The lack of availability indicates that regulatory tools are still insufficient or underutilized and have not yet resulted in quantifiable improvements in on-the-ground access for sheep and goats. Multisectoral approaches and complementary pathways are needed to introduce incentives for limited markets and to encourage the development, production and availability of essential veterinary medicines. In our survey-based study, clostridiosis and pasteurellosis vaccines, mastitis antibiotics, NSAID, and anthelmintics were the most frequently mentioned medicines, both in the context of their importance and unavailability for small ruminants.

Clostridial vaccines were reported as unavailable in some countries due to the lack of authorization, insufficient volume and frequency of market supply, and inadequate product volume and/or composition. Proposed solutions to increase availability include the authorization of clostridial vaccines in non-covered countries, increased trade and market supply, and the development of VMPs with smaller content volumes which could be optimal for the treatment of smaller herds. For pasteurellosis vaccines, in addition to the suboptimal market supply and lack of authorization in some countries, a common problem is that the composition of authorized *Pasteurella* vaccines does not adequately match the species or strains circulating in some countries. Often, the available VMPs do not provide protection against the most prevalent strains in affected countries. Moreover, certain countries have no access to stand-alone pasteurellosis vaccines but are limited to VMPs also containing clostridial components. Monitoring circulating strains, together with pharmacovigilance data from authorized products, is essential for assessing the suitability of available products. The RFSA Cartography of Therapeutic Gaps in France encountered similar problems for which the audited veterinarians cited field alternatives and practices [15].

Intramammary antibiotics are an unmet need in many EU countries for sheep, and in all for goats. Even at present, intramammary antibiotics are problematic for use due to increasing regulatory obstacles concerning prophylactic antibiotic use. “Cascade use” of products authorized for cattle is an unsatisfactory solution due to increased withdrawal periods. The RFSA Cartography additionally reminded of the good practice principles of early parenteral antibiotic therapy in clinical mastitis during lactation for animal welfare safeguarding, and immediate culling consideration. NSAIDs are an unmet need in all EU countries. As such, practitioners working with small ruminants have no alternative to “cascade use” of veterinary medicines authorized for other species. This survey study reveals multiple barriers to this option, such as long withdrawal periods and restriction of such use in organic farming in some countries. Other anti-inflammatory medicines, such as corticosteroids, are frequently not a viable alternative due to the absence of authorized VMPs in many countries, especially for sheep. Authorization on a central or national level is of utmost importance.

The limited market offer of antiparasitic medicines is a common problem in many EU countries. Veterinarians report a lack of efficacy in many individual APIs, which arises mostly due to the inability of veterinarians to rotate APIs and promote prudent and responsible antiparasitic use [45]. This regularly results in antiparasitic resistance [50]. The “cascade use” of anthelmintics authorized for other ruminants is in some countries only possible by presenting proof of resistance to available medicines which further complicates prudent and responsible use. The French initiative also emphasizes the responsibility associated with its use, which is in alignment with instructions and guidelines and promotes the careful and rational application of veterinary medicines to ensure both animal health and public safety. A common challenge reported for multiple countries is the complex procedure for interventional trade with other EU countries due to overwhelming bureaucratic burden and low possibility of approval. As such, interventional import or trade are not viable options for many countries and medicines.

## 5. Conclusions

The findings of this survey-based study provide empirical support for the premise that VMP shortages and unmet needs for sheep and goats are structural outcomes of market dynamics. Across countries, shortages, lack of availability, and unmet needs were most frequently reported in four critical categories: antimicrobials (particularly intramammary and systemic-use products); anti-inflammatory medicines, analgesics and antipyretics; antiparasitics, including ectoparasiticides VMPs; and vaccines, especially for endemic and production-limiting diseases. While approximately five thousand VMPs are authorized for sheep and goats at the EU level, only a limited proportion (n = 1450) are marketed and consistently accessible to practitioners. The EKE indicates that reported availability issues are rarely attributable solely to temporary supply disruptions. The VetMed+ Compass approach identified four highest-priority lack of availabilities for sheep, two for goats, as well as 13 highest-priority unmet needs for sheep and 14 for goats. The Compass aims to facilitate more targeted interventions, such as potentially incentivizing market entry, supporting cross-border prescription flexibility where appropriate, and guiding innovation toward high-priority unmet needs. Future research should focus on further specification of medicines within the medicine categories which are identified as a high priority in our survey study, since this allows for better tailored and actionable solutions. Additionally, due to the dynamic nature of these problems, further periodic quantitative research is needed to corroborate and refine the priorities identified in this exploratory study.

## Figures and Tables

**Table 1:** Categorization of veterinary medicine availability issues. This table categorizes the main definitions regarding lack of availability of VMPs. It distinguishes between products that are authorized, marketed, and available on the market, and provides typical causes for each situation.

Category	Authorized	Marketed	Available	Example Cause
(i) Shortage Based on HMA Good Practice Guidance for Communication to the Public on Medicines’ Availability Issues [12]	✅	✅	❌	Occurs when supply does not meet demand, often temporarily. Usually due to supply chain disruptions (due to, e.g., quality issues, manufacturing issues, unexpected increased demand)
(ii) Lack of Availability Based on EU Implementation Guide (Vet EU IG) on veterinary medicines product data in the Union Product Database [35]	✅	❌	❌	VMP is authorized but not marketed or accessible at a national, regional or EU level. Usually due to a MAH decision
(iii) Unmet need Based on Commission notice: Guidance to Applicants—Veterinary Medicinal Products (C/2024/1443) [29]	❌	❌	❌	A clinical need exists, but no authorized medicine is developed or authorized for the target species or clinical indication, at national or EU level

**Table 3:** Critically important antimicrobial medicine groups and subgroups. The table shows the reported critically important groups and subgroups of antimicrobial medicines along with the total number of mentions in the survey in descending order.

Antimicrobial Group	Antimicrobial Subgroup	Number of Mentions
General antibacterials	Antibiotics as a class (non-specified)	36
	Tetracyclines	28
	Narrow-spectrum penicillins, which are β-lactamase-sensitive.	19
	Macrolides, including combinations	17
	Aminopenicillins (without β-lactamase inhibitors)	13
	Aminoglycosides (except spectinomycin)	7
	Cephalosporins	6
	Quinolones (fluoroquinolones and other quinolones)	6
	Lincosamides	4
	Sulfonamides, dihydrofolate reductase inhibitors, and combinations	3
	Beta-lactam antibiotics, including combinations with β -lactamase inhibitors	3
	Antibacterial combinations	2
	Antibacterial–corticosteroid combinations	2
	Polymyxins	1
	Aminoglycosides (spectinomycin only)	1
	Amphenicols	1
Antibacterials for intramammary use	Non-specified	8
Antibacterials for topical and ocular use	Non-specified	3
	Anti-staphylococcal penicillins (beta-lactamase-resistant penicillins)	1
Antibacterials for genito-urinary use	Non-specified	1

**Table 4:** Anti-inflammatory medicine groups and subgroups. The table shows the reported critically important groups and subgroups of anti-inflammatory medicines along with the total number of mentions in the survey in descending order.

Medicine Group	Medicine Subgroup	Number of Mentions
Nonsteroidal anti-inflammatory drugs (NSAIDs)	Non-specified	27
	Oxicams	12
	Fenamates	6
	Propionic acid derivatives	2
Corticosteroids	Non-specified	9
	Glucocorticoid	9
Anti-inflammatory (non-specified)	Non-specified	14
Analgesics and antipyretics (non-specified)	Non-specified	4
Topical and ocular anti-inflammatory	Non-specified	3
Intramammary administration of anti-inflammatory	Non-specified	2
Other analgesics and antipyretics	Pyrazolones	1

**Table 5:** Critical groups and subgroups of antiparasitic medicines for small ruminants. The table shows the reported critically important groups and subgroups of antiparasitic medicines along with the total number of mentions in the survey in descending order.

Medicine Group	Medicine Subgroup	Number of Mentions
Anthelmintics	Non-specified	11
	Benzimidazoles	8
	Salicylanilides	3
	Imidazothiazoles/tetrahydropyrimidines	2
	Trematocides (non-specified)	2
	Amino-acetonitrile derivatives	2
	Combination of benzimidazole and related substances	1
Endectocides	Macrocyclic lactones	20
	Non-specified	1
Coccidiostats	Non-specified	6
	Coccidiostats (triazines)	3
	Coccidiostats (other)	1
Ectoparasiticides for topical use	Pyrethrins and pyrethroids	2
	Organophosphates	1
	Non-specified	1
Ectoparasiticides (non-specified)	Non-specified	3

**Table 6:** Critical groups and subgroups of vaccines for small ruminants. The table presents the critically important vaccine groups and subgroups, together with the total number of mentions, organized by category in a descending order.

Medicine Group	Medicine Subgroup	Number of Mentions
Bacterial vaccines	Clostridiosis/Enterotoxaemia	14
	Pasteurellosis	7
	Mastitis	6
	Chlamydiosis/Enzootic abortion of ewes (EAE)	6
	Foot rot	3
	Contagious agalactia	3
	Q fever	1
	Paratuberculosis	1
	Mycoplasmosis	1
Viral vaccines	Contagious ecthyma	1
Parasitic vaccines	Toxoplasmosis	2
Vaccines (other)	Not specified	6
	Respiratory disease (non-specified)	4
	Infections of the reproductive/obstetric system (non-specified)	2
	Combination	1

**Table 7:** European Veterinary Medicines Compass summary of shortages and lack of availability for sheep and goats. This table contains all highest priority veterinary medicines mentioned in the survey along with country tags, root causes and actionable solutions, as attributed by expert knowledge elicitation (EKE). Countries are tagged according to the ISO codes. Root causes are categorized according to Table 2 as follows: distribution issues and shortages (Dist), regulatory (Reg), unsatisfactory efficacy or safety (Efs.), absence of suitable VMP form (Form). Text in red color highlights obstacles in implementation of solutions. Vaccines which may need multivalent forms are marked with +.

Medicine Category	Medicine Group	Medicine Subgroup	Active Pharmaceutical Ingredient	Countries in Which the Medicine Is a Top Priority for Sheep	Countries in Which the Medicine Is a Top Priority for Goats	Root Cause Tag for Sheep (Country)	Root Cause Tag for Goats (Country)	Solutions for Sheep	Solutions for Goats
Antiparasitic medicines	Anthelmintics	Benzimidazoles	Albendazole	DK		Dist. (DK)		“Cascade use”	Dosages for “cascade use” should be available.
		Anthelmintics in general	General lack (Not specified)		ES, NL		Efs. (NL) Dist. (SE)	Prudent antiparasitic use/rotation of antiparasitic classes.	
								Registration of single API products. Use of combination products only prevents rotation of API	
								“Cascade use” Dosages for “cascade use” in goats should be available.	
								Vaccine development	
								Better market supply In some countries, veterinarians are not allowed to sell medicines so they rely on pharmacies to keep in stock.	
	Endectocides	Macrocyclic lactones	Moxidectin	DK		Dist. (DK)		“Cascade use”	Dosages for “cascade use” should be available.
Vaccines	Bacterial vaccines	Pasteuroellosis vaccine+	*Pasteurellacae* spp.	DE	FR, DE	Efs. + Unsuit. (DE)	Dist. + Efs. + Unsuit. (FR, DE)	Registration of specific species and strains vaccines Combined vaccines often lack the needed strain	
								Development of autogenous vaccines Sampling and pharmacovigilance must be regular	
								Pure Pasteurella vaccine without clostridial component is needed	
								“Cascade use” “Cascade use” officially not possible for vaccines in some countries	
								Increase the market supply	
	Viral vaccines	Bluetongue disease vaccines+	Multistrain bluetongue virus BTV	BE		Unsuit. (BE)		Improve trade and import schedule	
								Registration of multivalent vaccines Monovalent exist but are not practical	
								Modular principle vaccines with different serotypes which may be freely combined are highly necessary	
								Repellents help fight the spread of the disease via vectors, but existing products act as insecticides without repellent properties	

**Table 8:** European Veterinary Medicines Compass summary of unmet needs for sheep and goats. This table contains all highest priority veterinary medicines mentioned in the survey along with country tags, root causes and actionable solutions, as attributed by expert knowledge elicitation. Countries are tagged according to the ISO codes. Root causes are categorized according to Table 2 as follows: unmet needs on national level (UnmetNat), unmet needs on EU level (UnmetEU). Text in red color highlights obstacles in implementation of solutions. Vaccines which may need multivalent forms are marked with +.

Medicine Category	Medicine Group	Medicine Subgroup	Active Pharmaceutical Ingredient	Countries in Which the Medicine Is a Top Priority for Sheep	Countries in Which the Medicine Is a Top Priority for Goats	Root Cause Tag for Sheep (Country)	Root Cause Tag for Goats (Country)	Solutions for Sheep	Solutions for Goats
Antibacterial	Antibacterials for local use	Antibacterials for intra-mammary use	Clinical mastitis injectors (non-specified)		NL		UnmetNat		“Cascade use” Trade from BG, GR, FR
Anti-inflammatory	Nonsteroidal anti-inflammatory drugs	NSAIDs in general	General lack (Not specified)	NL		UnmetEU		“Cascade use” Withdrawal period too long in “cascade use” Not possible in organic farming in some countries	
Antiparasitic	Anthelmintics	Benzimidazoles	Netobimin	ES		UnmetNat		Oral suspension authorized in IT	
		Imidazothiazoles/Tetrahydro- pyrimidines	Levamisole as single ingredient VMP		DE		UnmetEU	Registration	
								Interventional purchase from BU, DE, ES, FR, GR, i.e., RO	
								Rotation of medicine groups.	
								Resistance present in alternatives.	
								“Cascade use” “Cascade use” in some countries is possible with presented proof of resistance to eprinomectin	
	Sex hormones, modulators of the genital system, and other gynecologicals	Sympathomimetics, labor repressants	Clenbuterol	DE		UnmetEU		Use of clenbuterol explicitly forbidden in sheep and goats in some countries	
	Dermatologicals	Antifungals for dermatological use	Antifungals for dermatomicosis in general (not specified)	ES		UnmetEU		Use of quaternary ammonium or sulfur oil	
		Foot rot treatment	Zinc sulfate bath	DE		UnmetEU		Use of alternatives: formalin, copper bath Copper concentration too low in existing products	
Vaccines	Bacterial	Clostridiosis/enterotoxemia+	*Clostridium haemolyticum*		DE		UnmetNat	Registration Current formulation product volume not suitable for use in small farms	
			*Clostridium tetani*					Trade	
			*Clostridium septicum*					Increased market supply	
			*Clostridium novyi*						
			*Clostridium perfringens* (Types A, B, C, and D)					“Cascade use” Officially not possible for vaccines in some countries	
			*Clostridium chauvoei*						
		Dichelobacter/footrot vaccines	*Dichelobacter nodosus*; *Fusobacterium necrophorum*	HR		UnmetNat		Registration Purchase from AT, BE, CZ, DE, ES, FR, i.e., IT NL, PT, SK Epidemiological studies for confirmation of disease prevalence are needed	
								Autogenous vaccines development	
		Paratuberculosis vaccines/Johne’s disease	*Mycobacterium avium* subspecies *paratuberculosis*	DE		UnmetNat		Purchase from GR, PT, ES, IS, NO, NL Special permission and written report after purchase or trade use needed in some countries	
		Mycoplasmosis/contagious agalactia vaccines +	*Mycoplasma agalactiae*		FR	UnmetNat		Purchase from BG, ES, RO, GR	
								Autogenous vaccines development	
		Caseous lymphadenitis vaccines	*Corynebacterium pseudotuberculosis*		HR		UnmetEU	Trade	
								Autogenous vaccine Efficacy of autogenous vaccines is low	
								Central registration	
		Listeriosis vaccines	*Listeria monocytogenes* (+*Listeria ivanovii*)	DE		UnmetEU		Vaccine research and development	
	Viral vaccines	Contagious ecthyma vaccines (Orf)	Parapoxvirus	ES		UnmetNat		Pharmacovigilance of the existing products	
								Echtymatisation	
								Purchase from FR Special permission and written report after import or trade use needed in some countries	
								Authorization of previously existing vaccines	
	Parasitic vaccines	Coccidiosis vaccines	Not specified	ES		UnmetEU		Development	
								Authorization	
		Cryptosporidiosis vaccines	*Cryptosporidium* spp.	FR		UnmetEU		Development	
								Authorization	
								Use of paromomycin, halofuginone, or immunoglobulins for treatment Paromomycin authorization for this indication must be expanded	

## Data Availability

The data presented in this survey based study were retrieved from the EMA Union Product Database in January and February 2026, last accessed on 11 February 2026. Availability data for veterinary medicinal products (VMPs) is based on the EMA Union Product Database (UPD) as reported by the Marketing Authorisation Holders (MAHs); however, this regulatory status may not accurately reflect actual field availability due to potential reporting delays The EMA Union Product Database is available at https://medicines.health.europa.eu/veterinary/en (last accessed on 11 February 2026).

## Supplementary Materials

The following supporting information can be downloaded at: https://www.mdpi.com/article/10.3390/vetsci13030297/s1, Figure S1: EKE reported frequency of medicine group availability lack; File S1: Survey form Table S1: STROBE statement; Table S2: Checklist for Reporting Results of Internet E-Surveys (CHERRIES); Table S3: Methods of LLT formation; Table S4: Chi-squared and Fisher exact test; Table S5: Complete EU Small Ruminants Compass.

## Abbreviations

The following abbreviations are used in this manuscript:

VMPs	Veterinary medicinal products
EKE	Expert knowledge elicitation
MAH	Marketing authorization holder
NSAIDs	Nonsteroidal anti-inflammatory drugs
UPD	Union Product Database
EU	European Union
AMR	Antimicrobial resistance
API	Active Pharmaceutical Ingredient
FVE	Federation of Veterinarians of Europe
EMA	European Medicines Agency
HERA	European Commission’s Health Emergency Preparedness and Response Authority
CMDv	Coordination Group for Mutual Recognition and Decentralized Procedures for Veterinary Medicinal Products
CVMP	Committee for Veterinary Medicinal Products
EEA	European Economic Area
HMA	Heads of Medicines Agencies
MS	Member State
SPOC	Medicine Shortages Single Point of Contact Working Party
WGCP	Working Group of Communication Professionals
WVA	World Veterinary Association
WSAVA	World Small Animal Veterinary Association
WOAH	World Organization for Animal Health
CAs	Competent authorities
PDO	Protected Designation of Origin
Vet EU IG	EU Implementation Guide
PPR	“Peste des petits ruminants”
EU MS	European union Member State
STROBE	Strengthening the Reporting of Observational Studies in Epidemiology
CHERRIES	Checklist for Reporting Results of Internet E-Surveys
EBVS	European Board for Veterinary Specialization
ECSRHM	European College of Small Ruminant Health Management
LLT	Lowest-level term
MRL	Maximum residue limit
RFSA	Le Réseau Français pour la Santé Animale
AMEG	Antimicrobial Advice Ad Hoc Expert Group
SPC	Summary product characteristics

## References

[1] World Health Organisation (WHO) (2022). One Health Joint Plan of Action (2022–2026): Working Together for the Health of Humans, Animals, Plants and the Environment. https://www.Who.Int/Publications/i/Item/9789240059139.

[2] World Organisation for Animal Health (WOAH) (2015). Animal Health: A Multifaceted Challenges. https://www.scribd.com/document/546120546/ANIMAL-HEALTH-EN-FINAL.

[3] European Commission (2022). Vulnerabilities of the Global Supply Chains of Medicines—Structured Dialogue on the Security of Medicines Supply. https://Health.Ec.Europa.Eu/Latest-Updates/Staff-Working-Document-Vulnerabilities-Global-Supply-Chains-Medicines-Structured-Dialogue-Security-2022-10-17_en.

[4] (2024). Coordination and Harmonization of the Existing Systems Against Shortages of Medicines—European Network (CHESSMEN). Analysis Report on Root-Causes. https://www.ja-chessmen.eu/files/upload/ficheiros/analysis-report-on-root-causes-d5-3.pdf?20250612093155.

[5] Whitehead M. (2012). Control of off-label use of medicines. Vet. Rec..

[6] Šandor K., Fajdić D., Pehnec M., Sinković S., Vretenar Špigelski K., Perak Junaković E., Vujnović A., Žarković I., Andrišić M., Terzić S. (2020). Mogućnost primjene neodobrenih veterinarsko-medicinskih proizvoda (off-Label Use). Vet. Stn..

[7] Tomanic D., Stojanovic D., Belić B., Davidov I., Novakov N., Radinovic M., Kladar N., Kovacevic Z. (2021). Off label use of human approved drugs in treatment of dogs in the Republic of Serbia. Pol. J. Vet. Sci..

[8] Federation of Veterinarians of Europe (FVE) (2022). Short Survey on Medicines/Vaccine Shortages. https://www.tieraerzteverband.de/bpt/aktuelles/meldungen/Dokumente/2022_11_08_FVE-Umfrage-TAM-Lieferengpaesse.pdf.

[9] Mottet A., Teillard F., Boettcher P., De’ Besi G., Besbes B. (2018). Review: Domestic herbivores and food security: Current contribution, trends and challenges for a sustainable development. Animal.

[10] Food and Agriculture Organization of the United Nations (FAO) Protecting People and Animals from Disease Threats. https://openknowledge.fao.org/server/api/core/bitstreams/9e1b3dfd-6a6a-4ecc-a2fc-7098e420b3cc/content.

[11] Charlier J., Barkema H.W., Becher P., De Benedictis P., Hansson I., Hennig-Pauka I., La Ragione R., Larsen L.E., Madoroba E., Maes D. (2022). Disease control tools to secure animal and public health in a densely populated world. Lancet Planet. Health.

[12] Vidhamaly V., Bellingham K., Newton P.N., Caillet C. (2022). The quality of veterinary medicines and their implications for One Health. BMJ Glob. Health.

[13] Karesh W.B., Dobson A., Lloyd-Smith J.O., Lubroth J., Dixon M.A., Bennett M., Aldrich S., Harrington T., Formenty P., Loh E.H. (2012). Ecology of zoonoses: Natural and unnatural histories. Lancet.

[14] FishMedPlus Coalition (2017). Fish Diseases Lacking Treatment. Gap Analysis Outcome. https://fve.org/publications/fishmedplus/.

[15] Réseau Français Pour la Santé Animale (RFSA) (2024). Cartography of Therapeutic Gaps in France. https://www.Reseau-Francais-Sante-Animale.Net/Le-Rfsa/Cartographie-Des-Gaps-Therapeutiques/.

[16] Fortineau O., Carnat Gautier P. (2014). Disponibilité du médicament vétérinaire: Réalité, enjeux et perspectives. Bull. L’acad. Vét. Fr..

[17] European Parliament and European Council (2022). Regulation (EU) 2019/6 of the European Parliament and of the Council of 11 December 2018 on Veterinary Medicinal Products and Repealing Directive 2001/82/EC. https://eur-lex.europa.eu/legal-content/EN/TXT/PDF/?uri=CELEX:02019R0006-20220128.

[18] Nogueira R., Baptista C.J., Gonçalves L., Coelho A.C., Faustino-Rocha A.I., Regueiro Purriños M., Gonzalo-Orden J.M., Oliveira P.A. (2024). The veterinary medicinal products market supply gap: A practical insight based on the Regulation (EU) 2019/6. Rev. Ciênc. Agrovet..

[19] Critical Medicines Alliance (2024). Strategic Report of The Critical Medicines Alliance. https://health.ec.europa.eu/document/download/3da9dfc0-c5e0-4583-a0f1-1652c7c18c3c_en?filename=hera_cma_strat-report_en.pdf.

[20] Federation of Veterinarians of Europe (FVE) (2019). Veterinary Medical Product and Pharmacovigilance Database—FVE Input. https://Fve.Org/Publications/Veterinary-Medical-Product-and-Pharmacovigilance-Database-Fve-Input/.

[21] Heads of Medicines Agencies (HMA) and the European Medicines Agency (EMA) (2019). EMA Guidance on Detection and Notification of Shortages of Medicinal Products for Marketing Authorisation Holders (MAHs) in the Union (EEA). https://www.ema.europa.eu/en/documents/regulatory-procedural-guideline/guidance-detection-and-notification-shortages-medicinal-products-marketing-authorisation-holders-mahs-union-eea_en.pdf.

[22] Heads of Medicines Agencies (HMA) and the European Medicines Agency (EMA) (2024). Good Practice Guidance for Communication to the Public on Medicines’ Availability Issues. https://www.ema.europa.eu/en/documents/regulatory-procedural-guideline/good-practice-guidance-communication-public-medicines-availability-issues_en.pdf.

[23] European Medicines Agency (EMA) (2023). The European Regulatory System for Medicines. https://www.ema.europa.eu/en/documents/leaflet/european-regulatory-system-medicines_en.pdf.

[24] European Medicines Agency (EMA) (2025). Seizing Opportunities in a Changing Medicines Landscape—The European Medicines Agencies Network Strategy 2028. https://www.ema.europa.eu/en/documents/other/seizing-opportunities-changing-medicines-landscape-european-medicines-agencies-network-strategy-2028-final_en.pdf.

[25] World Veterinary Association (WVA) (2025). Essential Veterinary Medicines List for Food-Producing Animals. https://worldvet.org/wp-content/uploads/2024/06/EVML-print-version_22032024.pdf.

[26] The World Small Animal Veterinary Association (WSAVA) (2023). List of Essential Medicines for Cats and Dogs. https://wsava.org/wp-content/uploads/2023/11/2023-essential-medicines-for-cats-and-dogs.pdf.

[27] World Organisation for Animal Health (WOAH) (2024). List of Antimicrobial Agents of Veterinary Importance. https://www.woah.org/app/uploads/2021/06/202501-en-woah-trd-list.pdf.

[28] Spanish Agency for Medicines and Medical Devices (AEMPS) (2024). Availability of Antimicrobial Medicinal Products and Alternatives to Their Use. https://www.resistenciaantibioticos.es/sites/default/files/2024-09/Availability_of_antimicrobial_medicinal_products_and_alternatives_to_their_use.pdf.

[29] European Commission (2024). Commission Notice. Guidance to Applicants—Veterinary Medicinal Products. https://eur-lex.europa.eu/legal-content/EN/TXT/PDF/?uri=CELEX:52024XC01443.

[30] European Commission (2026). Sheepmeat and Goatmeat Detailed Information on Imports, Trade, Market Measures, Legal Bases, Market Monitoring and Committees for Sheepmeat and Goatmeat. https://Agriculture.Ec.Europa.Eu/Farming/Animal-Products/Sheepmeat-and-Goatmeat_en#:~:text=Documents-,Overview,A%20loss%20of%20consumer%20confidence.

[31] Vinci C. (2014). The EU Dairy Sector Main Features, Challenges and Prospects. https://www.europarl.europa.eu/thinktank/en/document/EPRS_BRI(2018)630345.

[32] Rossi R. (2017). The Sheep and Goat Sector in the EU Main Features, Challenges and Prospects. European Parliamentary Research Service. https://www.europarl.europa.eu/thinktank/en/document/EPRS_BRI(2017)608663.

[33] Rossi R. (2018). The Future of the EU’s Sheep and Goat Sector. European Parliamentary Research Service. https://www.europarl.europa.eu/thinktank/en/document/EPRS_ATA(2018)620242.

[34] European Federation of Pharmaceutical Industries Associations (EFPIA) (2019). Addressing the Root Causes of Medicines Shortages. Supply Chain Stakeholders’ Views on Root Causes and Solutions. https://www.Efpia.Eu/Media/413378/Addressing-the-Root-Causes-of-Medicines-Shortages-Final-051219.Pdf.

[35] European Medicines Agency (EMA) (2025). The EU Implementation Guide (Vet EU IG) on Veterinary Medicines Product Data in the Union Product Database. https://www.ema.europa.eu/en/documents/regulatory-procedural-guideline/eu-implementation-guide-ig-veterinary-medicines-product-data-union-product-database-chapter-2-format-electronic-submission-veterinary-medicinal-product-information_en.pdf.

[36] Von Elm E., Altman D.G., Egger M., Pocock S.J., Gøtzsche P.C., Vandenbroucke J.P. (2008). The strengthening the reporting of observational studies in epidemiology (STROBE) statement: Guidelines for teporting observational studies. J. Clin. Epidemiol..

[37] Eysenbach G. (2004). Improving the quality of Web surveys: The checklist for reporting results of Internet E-surveys (CHERRIES). J. Med. Internet Res..

[38] Baduel L. (2024). Règlementation du médicament vétérinaire: Quel impact sur l’arsenal yhérapeutique à la disposition du praticien, en termes de disponibilité et d’innovation?. Bull. De L’académie Vétérinaire De Fr..

[39] World Organisation for Animal Health (WOAH) (2019). Priority Animal Diseases Sheets. Animal Health Pedagogical Toolkit. https://rr-africa.woah.org/app/uploads/2022/04/animal-health-educational-toolkit_priority-animal-diseases-sheets.pdf.

[40] European Medicines Agency (EMA) (2025). Categorisation of Antibiotics in the European Union. https://www.ema.europa.eu/en/documents/report/categorisation-antibiotics-european-union-answer-request-european-commission-updating-scientific-advice-impact-public-health-animal-health-use-antibiotics-animals_en.pdf.

[41] Crawford P.E., Hamer K., Lovatt F., Behnke M.C., Robinson P.A. (2023). Improving analgesia provision for sheep: An analysis of farm medicine records and attitudes towards pain relief on sheep farms in Northern Ireland. Vet. Rec. Open.

[42] Tsekouras N., Christodoulopoulos M.A.B., Meletis E., Kousoulis C., Kostoulas P., Pantazis V., Papatsiros V.G., Dimoveli K., Gougoulis D. (2025). Associations between non-steroidal and steroidal anti-inflammatory drug use, Welfare, and Milk Production in Dairy Sheep: A Multivariate Study. Animals.

[43] Anadón A., Ares I., Martínez-Larrañaga M.R., Martínez M., Martínez M.-A., Ramawat K.G., Mérillon J.-M. (2025). Pyrethrins and pyrethroids: Ectoparasiticide use in veterinary nedicine. Natural Products.

[44] Rose H., Rinaldi L., Bosco A., Mavrot F., de Waal T., Skuce P., Charlier J., Torgerson P.R., Hertzberg H., Hendrickx G. (2015). Widespread anthelmintic resistance in European farmed ruminants: A Systematic review. Vet. Rec..

[45] World Organisation for Animal Health (WOAH) (2022). Responsible and Prudent Use of Anthelmintic Chemicals to Help Control Anthelmintic Resistance in Grazing Livestock Species. https://www.woah.org/app/uploads/2021/12/oie-anthelmintics-prudent-and-responsible-use-final-v4-web-opt.pdf.

[46] Altuğ N., Özdemir R., Cantekin Z. (2013). Preventive medicine in ruminants: I. Vaccination program. Erciyes Üniv. Vet. Fak. Derg..

[47] Serhan Serhat A.Y. (2017). Abortion Problems and reproductive vaccination programs in small ruminants. Turkiye Klinikleri J. Vet. Sci. Obs. Gynecol-Spec. Top..

[48] European Commission (2020). Pharmaceutical Strategy for Europe. https://Health.Ec.Europa.Eu/Medicinal-Products/Pharmaceutical-Strategy-Europe_en.

[49] Anadón A., Martínez-Larrañaga M.R., Ares I., Martinez M.A. (2025). Regulatory aspects for the Drugs and Chemicals Used in Food-Producing Animals in the European Union. Veterinary Toxicology Basic and Clinical Principles.

[50] Food and Agriculture Organization of the United Nations (FAO) (2004). Guidelines Resistance Management and Integrated Parasite Control in Ruminants. https://openknowledge.fao.org/server/api/core/bitstreams/8efa816b-a7d5-4667-8c33-777fd35bc13b/content.

