# Supplementary material for: Development of the European Veterinary Medicines Gaps and Needs Compass for Sheep and Goats Based on Online Survey and Expert Knowledge Elicitation
Source: Vet Sci. 2026 Mar 21;13(3):297. doi: 10.3390/vetsci13030297 (PMC13030040; doi:10.3390/vetsci13030297)
Supplement: Supplementary file 1 [file vetsci-13-00297-s001.zip › Supplementary figure S1_EKE reported frequency of medicine group availability lack .pdf]

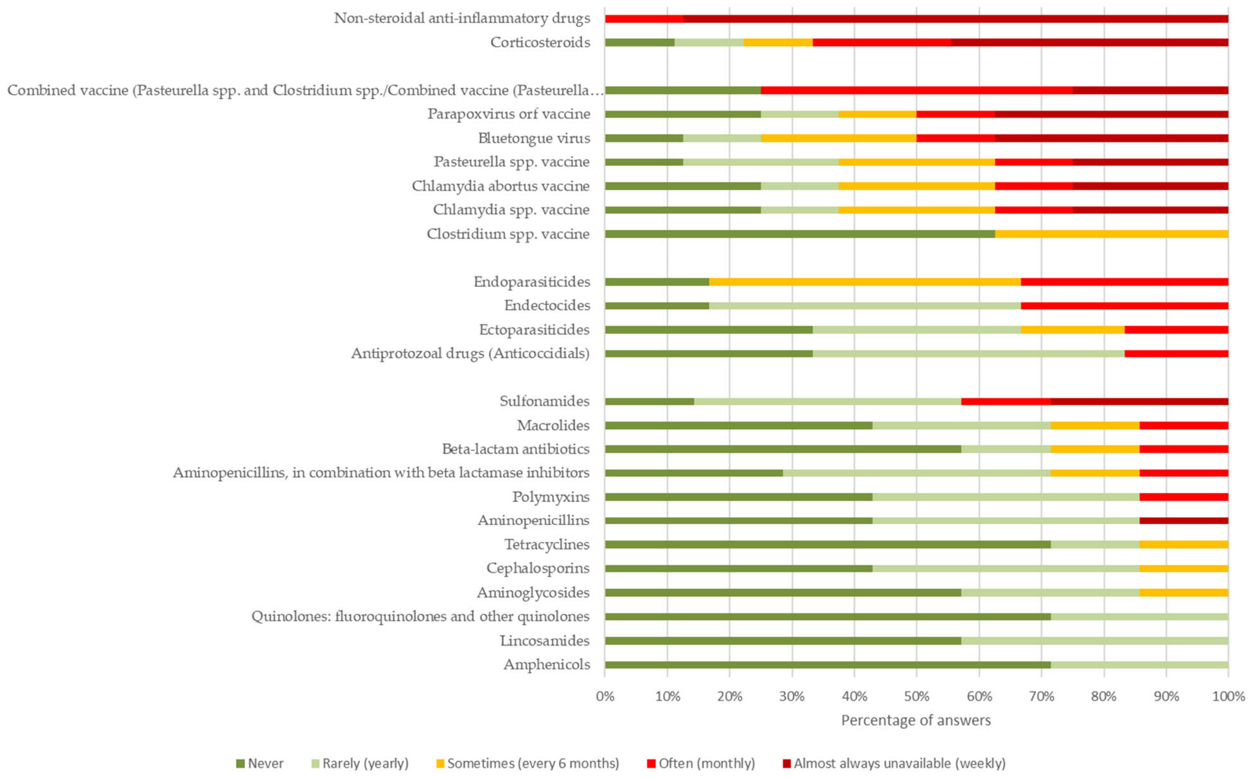


Supplementary Figure S1 Reported frequency of medicine group availability lack for sheep and goat. The figure shows the frequency of the lack of availability per medicine groups in descending order among reported medicine categories in the survey study, as identified by EKE.
