# Supplementary material for: Development of the European Veterinary Medicines Gaps and Needs Compass for Sheep and Goats Based on Online Survey and Expert Knowledge Elicitation
Source: Vet Sci. 2026 Mar 21;13(3):297. doi: 10.3390/vetsci13030297 (PMC13030040; doi:10.3390/vetsci13030297)
Supplement: Supplementary file 1 [file vetsci-13-00297-s001.zip › Supplementary file S1_Survey form.pdf]

FVE list of essential medicines for Sheep and Goats

Many European countries face critical lack of availability of veterinary medicines, particularly for minor species and minor uses. Some medicines are authorised and on the market in other countries, while others are entirely lacking. These gaps directly impact animal health, welfare, veterinary public health and the profitability of farmers.

The FVE/UEVP Medicines Working Group is **mapping essential veterinary medicines across Europe**. We are starting with sheep and goats. We thank you all to offer your support as small ruminants experts from different European countries!

In particular, FVE is developing Essential Medicines Lists, categorised by species and by country. These lists will identify key medicines that should be available for veterinary use and guide marketing authorisation holders and authorities to address availability issues. It will also build on existing references like the WVA Essential Veterinary Medicines List forfood-producing animals and WSAVA List of Essential Medicines for Cats and Dogs and France's RSFA cartography of therapeutic gaps.

In this short survey, we would like to gather initial field experience in regard of the use of medicines for sheep and goats. This information will guide the work further along.

We aim to gain information on the **most frequent and impactful diseases for small ruminants** in respect to animal health, welfare, public health and economic impact in your country, to investigate possible **therapeutic gaps** and **shortages,** and to **identify essential medicines** that practitioners in the field of small ruminants could not live without.

Data will be handled anonymously and in line with the EU GDPR rules.

For questions please contact Marine Delsaute, FVE (marine@fve.org)

* Indicates required question

1. 1. In which country do you work as a veterinarian? * Dropdown

Mark only one oval.

- Albania
- Armenia
- Austria
- Belgium
- Bosnia/Herzegovina
- Bulgaria
- Croatia
- Cyprus
- Czech Republic
- Denmark
- Estonia
- Finland
- France
- Germany
- Greece
- Hungary
- Iceland
- Ireland
- Italy
- Latvia
- Lithuania
- Luxembourg
- Malta
- Montenegro
- Netherlands
- North Macedonia
- Norway
- Poland
- Portugal
- Romania
- Serbia
- Slovakia

- Slovenia
- Spain
- Sweden
- Switzerland
- Ukraine
- United Kingdom
- Other

2. 2. How many years of experience do you have as a veterinarian? *

Mark only one oval.

- <5 years
- 6-15 years
- 16-25 years
- >25 years

3. 3. In which type of practice or workplace do you work? *

Mark only one oval.

- Mixed practice
- Practice specialised in sheep and goats
- Practice specialised in Ruminants
- Academia and research
- Industry
- State Veterinary Medicine
- Other: ______________________

4. 5. Based on your experience, what are the most common diseases/indications affecting sheep and goats in your country? Please list up to 10 diseases/indications.

5. 6. In your opinion, are there notable therapeutic gaps in respect to medicines or vaccines for sheep and goats in your country? If yes, please specify them.

6. 7. As a veterinarian working with sheep and goats, what medicines would you consider critical in your dialy practice? Please list up to 10.

7. 8. Do you often face lack of availability of veterinary medicines (e.g. due to supply chain issues an authorised medicine is not available) for sheep and goats in your country (e.g. due to supply chain issues an authorised medicine is not available)? If so, how often and which medicines mainly?

8. 9. Do you have any further insights or recommendations that could contribute to the development of an essential medicines list for sheep and goats?

9. 10. Do you have any resources you would like to share that could support the development of an essential medicines list for sheep and goats?

10. 11. If you would like to receive the final results of this survey, please leave your email address below.

This content is neither created nor endorsed by Google.

Google Forms
