# Supplementary material for: Development of the European Veterinary Medicines Gaps and Needs Compass for Sheep and Goats Based on Online Survey and Expert Knowledge Elicitation
Source: Vet Sci. 2026 Mar 21;13(3):297. doi: 10.3390/vetsci13030297 (PMC13030040; doi:10.3390/vetsci13030297)
Supplement: Supplementary file 1 [file vetsci-13-00297-s001.zip › Supplementary table S3_Methods of LLT formation .pdf]

Supplementary table S3. Methods of LLT formation according to the survey section. The table shows the method of common category assignment in the formation of LLT by each type of information given in the survey

Information given		Method of common category assignment
**Demographic data**	**Years of experience as a veterinarian**	<5 years; 6–15 years; 16–25 years; >25 years
	**Workplace**	Division based on clinical and non-clinical workplace roles
**Disease burden**		• Infectious / non-infectious<br>• Organ system<br>• Etiology + organic system
**Critical medicines**		Names of classes were chosen mostly based on the Anatomical, Therapeutical and Chemical (ATCVet) code, with necessary modifications for non-aligning answers.<br>• Category<br>• Group<br>• Subgroup<br>• Active Pharmaceutical Ingredient (API)
**Lack of availability**		• Mentioned species<br>• Mentioned medicine (according to "critical medicines" criteria)<br>• Frequency
**Unmet needs**		• Diseases mentioned (according to "disease burden" criteria)<br>• Mentioned medicine (according to "critical medicines" criteria)
