# Supplementary material for: Development of the European Veterinary Medicines Gaps and Needs Compass for Sheep and Goats Based on Online Survey and Expert Knowledge Elicitation
Source: Vet Sci. 2026 Mar 21;13(3):297. doi: 10.3390/vetsci13030297 (PMC13030040; doi:10.3390/vetsci13030297)
Supplement: Supplementary file 1 [file vetsci-13-00297-s001.zip › Supplementary table S4_Chi-squared and Fisher exact test .pdf]

Supplementary table S4. Chi-squared and Fisher exact test results for categories of mentioned medicines as Lack of availability. Statistically significant values are marked with *

**Medicine category**	**Degrees of freedom, sample size, p value**
Vaccines	X2 (1, N = 151) = 4.96, p = .026*
Antimicrobial medicines	(N = 151), p = .029*
Antiparasitic products, insecticides and repellents	(N = 151), p = .087
Other categories	(N= 151), p = .041*
