# Supplementary material for: Development of the European Veterinary Medicines Gaps and Needs Compass for Sheep and Goats Based on Online Survey and Expert Knowledge Elicitation
Source: Vet Sci. 2026 Mar 21;13(3):297. doi: 10.3390/vetsci13030297 (PMC13030040; doi:10.3390/vetsci13030297)
Supplement: Supplementary file 1 [file vetsci-13-00297-s001.zip › Supplementary table S5_Complete EU Small Ruminants Compass.pdf]

Supplementary table S5. European Veterinary Medicines Compass summary of unmet needs, shortages and lack of availability for sheep and goats. This table contains all veterinary medicines mentioned in the survey and expert knowledge elicitation (EKE) along with priority levels, country tags, root causes and actionable solutions, as attributed by expert knowledge elicitation. Countries are tagged according to the ISO codes. Root causes are categorized according to Table 2 as follows: Unmet needs on national level (UnmetNat), Unmet needs on EU level (UnmetEU) Distribution issues and shortages (Dist), Regulatory (Reg). Perceived insufficient efficacy or safety (Efs.), Absence of suitable VMP form (Form). Level of priority is assigned as follows: "very urgent, issue must be solved within next 3 years" (priority 1), "urgent" (priority 2), "needed soon" (priority 3), "can be solved by the "Cascade use"" (priority 4), "not a priority, authorized medicine exists for the species" (priority 5). Text in red color highlights obstacles in implementation of solutions. vaccines which may need multivalent forms are marked with +. Data acquired exclusively by the EKE is marked with *.

Medicine group	Medicine subgroup	Active pharmaceutical ingredient / etiology	Priority for sheep (Country)	Priority for goats (Country)	Root cause tag for sheep (Survey/EKE Country)	Root cause tag for goats (Survey/EKE Country)	Country specific solution for sheep	Country specific solution for goats	Authorised where for sheep (any life stage) according to UPD	Where is availability according to UPD in January 2026 (sheep)	Root cause tag for sheep according to UPD (January 2026)	Authorised where for goats (any life stage) according to UPD	Where is availability according to UPD in January 2026 (goats)	Root cause tag for goat according to UPD (January 2026)
Bacterial vaccines	Clostridiosis / enterotoxemia vaccines+	*Clostridium haemolyticum*	1 (HR*), 5 (ES, SE, DE)	1 (HR*), 5 (ES), 3 (DE*), 4 (SE)	UnmetNat + Form. (HR*, DE*)<br>ES, IT, UK(NI)<br>Dist. (SE)	ES, IT, UK(NI), GR*	Registration / Trade / import (HR*)<br>Current product volume not suitable for small farms<br>Increase the marketed amounts (SE)		CY, DE, FR, PT, PL, BG, SK, SI, IT, RO, IE, CZ, UK(NI), LU, NL, EE, LV, LT, BE, GR	PL, SK, CZ, UK(NI), LU, NL, EE, LV, LT, BE	UnmetNat	/	/	UnmetEU
		Clostridium sordellii	5 (ES, SE, DE)	5 (ES), 3 (GR*), 4 (SE)	UnmetNat + Form. (HR*)<br>ES, IT, DE, UK(NI)<br>Dist. (SE)	UnmetNat (HR*, GR*) + Form. (HR*)			BG, PT, LV, EE, UK(NI), SE, DE, LU, FI, NO, DK, SK, ES, RO, BE, IT, AT, IS, NL, LT, PL, CZ, CY, HU, FR, IE, GR, SI	BG, SE, DE, LU, FI, NO, DK, SK, PT, RO, BE, IT, AT, IS, NL, LT, PL, CZ, CY, HU, FR, IE, GR, ES	UnmetNat	BG, PT, ES, GR, NL, DE, AT	BG, ES, GR, PT	UnmetNat
		Clostridium tetani	1 (HR*), 5 (ES, SE, DE)	1 (HR*, DE*), 4 (SE), 5 (ES)	UnmetNat + Form. (HR*, DE*)<br>ES, IT, UK(NI), SE<br>Dist. (SE)	ES, IT, UK(NI), SE, GR*			BG, ES, IT, PT, PL, RO, SK, LV, HU, CZ, FR, IE, GR, UK(NI), IS, AT, NL, NO	BG, PL, GR, RO, IE, UK(NI), IT, PT, ES, IS, NL, NO	UnmetNat	BG, RO, SK, LV, HU, CZ, PL, FR, AT, PT, ES, GR	BG, PL, PT, ES	UnmetNat
		Clostridium septicum	1 (HR*), 5 (ES, SE, DE)	1 (HR*, DE), 4 (SE), 5 (ES)	UnmetNat + Form. (HR*)<br>ES, IT, DE, UK(NI)<br>Dist. (SE)	ES, IT, DE, UK(NI), GR*			BG, ES, LV, UK(NI), EE, SE, DE, LU, FI, NO, DK, SK, PT, RO, BE, IT, AT, IS, NL, LT, PL, CZ, CY, HU, FR, IE, GR, SI	BG, ES, SE, DE, LU, FI, NO, DK, SK, PT, RO, BE, IT, AT, IS, NL, LT, PL, CZ, CY, HU, FR, IE, GR, UK(NI), EE, LV	UnmetNat	BG, ES, FR, RO, GR, NL, DE, AT, PT	BG, ES, GR, PT	UnmetNat
		Clostridium novyi	1 (HR*), 5 (ES, SE, DE)	1 (HR*), 2 (DE) 4 (SE), 5 (ES)	UnmetNat + Form. (HR*)<br>ES, IT, DE, UK(NI)<br>Dist. (SE)	ES, IT, DE, UK(NI), GR*,			BG, ES, LV, UK(NI), EE, SE, DE, LU, FI, NO, DK, SK, PT, RO, BE, IT, AT, IS, NL, LT, PL, CZ, CY, HU, FR, IE, GR, SI	BG, ES, SE, DE, LU, FI, NO, DK, SK, PT, RO, BE, IT, AT, IS, NL, LT, PL, CZ, CY, HU, FR, IE, GR, UK(NI), EE, LV	UnmetNat	BG, ES, FR, RO, GR, NL, DE, AT, PT	BG, ES, GR, PT	UnmetNat
		Clostridium perfringens (Types A, B, C, and D)	1 (HR*), 5 (ES, SE, DE)	1 (HR*, DE*), 4 (SE), 5 (ES)	UnmetNat + Form. (HR*, DE*)<br>ES, IT, UK(NI)<br>Dist. (SE)	ES, IT, UK(NI), GR*			BG, GR, ES, PT, LV, UK(NI), EE, SE,DE, LU, FI, NO, DK, SK, PT, RO, BE, IT, AT, IS, NL, LT, PL, CZ, CY, HU, FR, IE, GR, SI	BG, ES, SE, DE, LU, FI, NO, DK, SK, PT, RO, BE, IT, AT, IS, NL, LT, PL, CZ, CY, HU, FR, IE, GR, UK(NI), EE, LV	UnmetNat	BG, ES, PT, RO, SK, HU, CZ, FR, GR, NL, DE, AT, PT	BG, ES, GR, PT	UnmetNat
		Clostridium chauvoei	1 (HR*), 5 (ES, SE, DE)	h	UnmetNat + Form. (HR*, DE*)<br>ES, IT, UK(NI)<br>Dist. (SE)	UnmetNat + Form. (HR*, DE*)<br>ES, IT, UK(NI), GR*			BG, ES, IE, UK(NI), LV, EE, SE, DE, LU, FI, NO, DK, SK, PT, RO, BE, IT, AT, IS, NL, LT, PL, CZ, CY, HU, FR, GR, SI	BG, ES, SE,DE, LU, FI, NO, DK, SK, PT, RO, BE, IT, AT, IS, NL, LT, PL, CZ, CY, HU, FR, IE, GR, UK(NI), EE, LV	UnmetNat	BG, ES, FR, RO, GR, NL, DE, AT, PT	BG, ES, GR, PT	UnmetNat
	Pasteuroellosis vaccine+	*Pasteurellacae* spp.	5 (ES), 2 (FR, GR, SE), 5 (DE)	5 (ES), 1 (FR), 2 (GR), 3 (SE, DE*)	Efs. + Form. (FR)<br>Dist. (GR, SE)<br>Form. (DE*)<br>ES, DE, IT, UK (NI)	Dist. + Efs. + Form. (FR)<br>UnmetNat (GR)<br>Dist. (SE)	Registration of specific *Pasteurella* species and strains vaccines<br>Combined vaccines often lack the needed strain<br>Development of autogenous vaccines<br>Sampling and pharmacovigilance must be regular (FR)<br>Increase the marketed<br>Pure vaccine without clostridial component needed in Germany	Cascade use (GR), Increase	/	/	UnmetEU	/	/	UnmetEU
		Pasteurella multocida	5 (ES)		ES				ES, BG, PT, FR	ES		ES, BG, PT, FR	ES	
		*Bibersteinia trehalosi**	5 (DE*)	3 (GR*)	UnmetEU	UnmetEU (GR*)		Cascade use (GR*)	RO, DE, IE, UK(NI), IT, PT, ES, BG, FR, GR, NO	RO, DE, IE, UK(NI), IT, PT, ES, FR, GR, NO	UnmetNat	/	/	UnmetEU
		Mannheimia haemolytica	2 (DE*), 5 (ES)		ES, UnmetNat (DE*)	ES, FR, UnmetNat (DE*)	Autogenous vaccines (DE*)<br>All serotypes needed		ES, PT, GR, BG, RO, DE, IE, UK(NI), IT, FR, NO	ES, GR, RO, DE, IE, UK(NI), IT, PT, FR, NO	UnmetNat	ES, PT, BG, FR	ES, FR	UnmetNat
	Mastitis vaccines+	*Staphylococcus aureus*	5 (ES, DE*)		ES, IT									
		Streptococcus agalactiae			UnmetEU				/	/	UnmetEU	/	/	UnmetEU
		Streptococcus dysgalactiae							/	/	UnmetEU	/	/	UnmetEU
		*Trueperella pyogenes**							BG	/	UnmetNat	/	/	UnmetNat
		Mastits vaccines in general (non-specified)			ES									
	Dichelobacter / footrot vaccines	*Dichelobacter nodosus; Fusobacterium necrophorum*	1 (HR), 5 (DE*)	3 (HR*, DE*)	UnmetEU (HR)		Registration (HR)<br>Epidemiological Serotype specific<br>Cascade use (DE*)<br>Cascade use officially not allowed in Germany<br>Multiple serotypes needed		BG, PT, BE, NL, AT, DE, IE, FR, ES, SK, CZ, IT	PT, BE, NL, AT, DE, IE, FR, ES, SK, CZ, IT	UnmetNat	BG	/	UnmetNat

	Chlamydiosis / Enzootic abortion of ewes (EWE) vaccines	*Chlamydophila abortus*	5 (ES, DE*), 2 (GR)	5 (ES), 2 (GR), 1 (DE*)	UnmetEU (ES, GR, UK(NI))		Increase the Trade / imported amounts / Registration (GR)	Cascade use (GR, DE*)<br>Cascade use officially not allowed in Germany	FR, ES, IT, IE, GR, CY, AT, DE, HU, LU, NL, PT, RO, UK(NI), BE, PL	IE, GR, CY, FR, ES, DE, IT, NL	UnmetNat	ES, IT	/	UnmetNat
	Q fever vaccine	*Coxiella burnetii*	5 (DE)		UnmetEU (DE, IT)				AT, BE, BG, HR, CY, CZ, DE, EE, FI, FR, DK, GR, HU, LU, IS, IE, IT, LV, LI, LT, MT, NL, NO, PL, PT, RO, SK, SI, ES, SE	/	Lack of availability	AT, BE, BG, HR, CY, CZ, DE, EE, FI, FR, DK, GR, HU, LU, IS, IE, IT, LV, LI, LT, MT, NL, NO, PL, PT, RO, SK, SI, ES, SE	/	Lack of availability
		Mycobacterium avium subspecies *paratuberculosis*	5 (ES), 1 (DE)	5 (ES, FR), 1 (DE)	ES, UnmetNat (DE)	ES, FR, UnmetNat (DE)			GR, PT, ES, IS, NO, NL, FR, IT	GR, PT, ES, IS, NO, NL	UnmetNat	GR, PT, ES, IS, NO, NL, FR, IT	GR, PT, ES, IS, NO, NL	UnmetNat
	Mycoplasmosis / contagious agalactia / contagious caprine pleuropneumonia (CCPP) vaccines +	*Mycoplasma agalactiae*	5 (ES), 2 (FR)	5 (ES), 1 (FR)	UnmetNat (ES, FR)		Trade / import / autogenous vaccine (FR)		BG, ES, RO, GR, PT, IT	BG, ES, RO, GR	UnmetNat	BG, ES, RO, GR, PT	BG, ES, RO, GR	UnmetNat
		Mycoplasma capricolum			UnmetNat (FR)				BG, ES, PT	BG, ES	UnmetNat	BG, ES, PT	BG, ES	UnmetNat
		Mycoplasma capricolum subsp. *Capripneumoniae**	1 (DE*)		UnmetNat (DE*)		Disease not yet present in Germany so no vaccine is marketed		/	/	UnmetEU	/	/	UnmetEU
		Mycoplasma mycoides			FR									
		*Mycoplasma ovipneumoniae**	3 (SE, GR*, DE*)		UnmetEU (GR*)		Research and development, Trade / import (SE)<br>Autogenous vaccines (DE*)		/	/	UnmetEU	/	/	UnmetEU
		Mycoplasma spp.												
	Caseous lymphadenitis vaccines	*Corynebacterium pseudotuberculosis*	1 (DE*), 3 (HR*), 3 (ES, NL), 4 (FR)	1 (HR*, NL, DE*), 3 (ES, FR)	UnmetEU (ES, FR, NL, DE*, HR*)		Trade / import / Autogenous vaccine (FR, DE*)<br>Efficacy of autogenous vaccines is low<br>Central registration / Trade / import (NL, HR*)		/	/	UnmetEU	/	/	UnmetEU
	Colibacillosis / Watery mouth vaccines	*Escherichia coli*	2 (FR)		UnmetEU (FR, UK(NI))		Registration / autogenous vaccine / zoohygienic measures (FR)		FR, PL	/	UnmetNat	/	/	UnmetEU
	Anthrax vaccines	*Bacillus anthracis*	5 (ES)		ES									
	Listeriosis vaccine	*Listeria monocytogenes*<br>*Listeria ivanovii*	1 (DE)		UnmetEU (DE)				/	/	UnmetEU	/	/	UnmetEU
	Other bacterial vaccines	Joint ill vaccine (non-specified)			UK(NI)									
	Other combination	*Pasteurella* spp.<br>*Clostridium* spp.			UnmetEU (SE)		Research and development (SE)		PT	/	UnmetNat	PT	/	UnmetNat
	Contagious ecthyma vaccines (Orf)	Parapoxvirus	1 (ES), 2 (DE*), 3 (FR, GR*)		Efs. (FR)<br>UnmetNat (ES, DE, GR*)		Pharmacovigilance of the existing products / Echtymatisation (FR)<br>Trade / import (ES, GR*, DE*)<br>Special individual permission needed in Germany<br>Reauthorisation of previously existing vaccines (ES)		FR, ES	FR	UnmetNat	FR, ES	FR	UnmetNat
		Multistrain bluetongue virus BTV	1 (BE, DE*), 5 (ES), 3 (GR*)	1 (DE*), 3 (BE), 5 (ES)	Dist. (GR*)<br>Form. (BE, DE*)	UnmetEU (ES, BE, GR*)	Improve Trade / import schedule (GR*)<br>Registration of multivalent vaccines (BE, DE*)<br>Monovalent exist but are not practical<br>Need of inactivated BTV vaccines of different serotypes, based on a modular principle, in which the epidemiologically necessary serotypes can be freely combined<br>Use of repellents (DE*)		HR, AT, BE, BG, CY, CZ, DK, EE, FI, FR, DE, GR, HU, IS, IE, IT, LV, LI, LT, LU, MT, NL, PL, NO, PT, RO, SK, SI, ES, SE, UK(NI)	FR, AT, IT, PT, ES, BE, DE		/	/	UnmetEU

Viral vaccines	Bluetongue disease vaccines+				ES		No authorized repellents available, only contact insecticides							
		Bluetongue virus BTV-1*	1 (HR*)		UnmetNat (HR*)	UnmetEU HR*	Epidemiological studies of prevalence / Trade / import (HR*)		HR, AT, BE, BG, CY, CZ, DK, EE, FI, FR, DE, GR, HU, IS, IE, IT, LV, LI, LT, LU, MT, NL, PL, NO, PT, RO, SK, SI,	ES		/	/	UnmetEU
		Bluetongue virus BTV-3				UnmetEU			HR, AT, BE, BG, CY, CZ, DK, EE, FI, FR, DE, GR, HU, IS, IE, IT, LV, LI, LT, LU, MT, NL, PL, NO, PT, RO, SK, SI, ES, SE, UK(NI)	FR, DK, ES, FI		/	/	UnmetEU
		Bluetongue virus BTV-4*	1 (HR*)		UnmetNat (HR*)	UnmetEU HR*	Epidemiological studies of prevalence / Trade / import (HR*)		HR, AT, BE, BG, CY, CZ, DK, EE, FI, FR, DE, GR, HU, IS, IE, IT, LV, LI, LT, LU, MT, NL, PL, NO, PT, RO, SK, SI, ES, SE, UK(NI)	GR, BE, DE, PT, SK, ES, RO, FR, IT		BG		UnmetNat
		Bluetongue virus BTV-5			UnmetEU				/	/	UnmetEU	/	/	UnmetEU
		Bluetongue virus BTV-8*	1 (HR*)		UnmetNat (HR*)	UnmetEU HR*	Epidemiological studies of prevalence / Trade / import (HR*)		HR, AT, BE, BG, CY, CZ, DK, EE, FI, FR, DE, GR, HU, IS, IE, IT, LV, LI, LT, LU, MT, NL, PL, NO, PT, RO, SK, SI, ES, SE, UK(NI)	FR, LU, IT, ES		/	/	UnmetEU
	Peste des Petits Ruminants vaccine (PPR)	*Morbilivirus**	1 (DE*)		UnmetEU	UnmetEU DE*	Disease not present in Germany so no vaccine available		/	/	UnmetEU	/	/	UnmetEU
	Rabies vaccines	*Lyssavirus rabies**	5 (DE*)		UnmetEU	UnmetEU DE*			SI, HU, EE, CY, LT, SK, BG, LV, RO, HR, ES, IT, RO, NL, BE, SE, PL, CZ, DE, IE, FR, PT, LU, FI, NO, DK, AT, GR, UK(NI)	ES, IT, RO, BG, SK, NL, BE, LV, HU, SE, PL, LT, CZ, HR, EE, DE, FR, LU, FI, NO, DK, AT, CY, GR, UK(NI), IE	UnmetNat	SI, HU, EE, CY, LT, SK, BG, LV, RO, HR, IT, RO, SE, PL, CZ, GR, UK(NI), BE, DK, DE, AT, FI, FR, IE, NL, NO, LU	IT, RO, BG, SK, LV, HU, SE, PL, LT, CZ, HR, EE, GR, UK(NI), BE, DK, DE, AT, FI, IE, NL, NO, CY, LU	UnmetNat
	Retroviral diseases	Not specified			UnmetEU (FR)						UnmetEU			UnmetEU
Parasitic vaccines	Toxoplasmosis vaccines	*Toxoplasma gondii*	1 (DE*), 3 (ES, GR)		UnmetEU (ES, GR, DE*)		Epidemiological studies of prevalence / need Trade from France (DE*) Special Trade / import license needed in Germany		ES, FR, NL, IE	ES, FR, IE	UnmetNat	/	/	UnmetEU
	Coccidiosis vaccines	Not specified	1 (ES*)		UnmetEU (ES)		Development / authorisation		/	/	UnmetEU	/	/	UnmetEU
	Cryptosporidiosis vaccine	*Cryptosporidium* spp.	1 (ES*, FR)		UnmetEU (ES*, FR)		Development / authorization / use of paromomycin, halofuginone, or immunoglobulins for treatment (ES*, FR) Paromomycin authorization for this indication must be expanded		/	/	UnmetEU	/	/	UnmetEU
	Sheep scab vaccines	*Psoroptes ovis*			UnmetEU (UK(NI))				/	/	UnmetEU	/	/	UnmetEU
	Haemonchosis vaccines (Barber's pole)	*Haemonchus contortus**	1 (DE*)		UnmetEU DE*		Research and development (DE*)		/	/	UnmetEU	/	/	UnmetEU
	Hemoparasites vaccines*	*Babesia* spp.*					Development of vaccines *Babesia* spp, (ES*)							
		Theileria spp. *			UnmetEU (ES*)		Development of vaccines *Theileria* spp., (ES*)							
		Anaplasma spp. *					Development of vaccines *Anaplasma* spp., (ES*)							
	Parasitic vaccines in general	Not specified			N/A (UK(NI))	UnmetEU (UK(NI))			ES, FR, NL, GR, IR	FR, IR, ES		/	/	UnmetEU
	Autogenous vaccines	*Mycoplasma* spp.	3 (FR)	1 (FR)	FR		Trade / import / autogenous vaccine development (FR)							
		Autogenous vaccines in general			FR									
		Neonatal diarrhea vaccines	2 (GR, NL)		N/A (GR, NL)		Cascade use (GR, NL)		N/A	N/A		N/A	N/A	
		Septicemia vaccines			ES									
		Abortion vaccines			ES									
		Reproductive system infections vaccines												

Other vaccines	Miscellaneous diseases	Respiratory infections vaccines	2 (GR)		Dist. (GR)	UnmetNat (GR)	Increase the Trade / imported amounts (GR)	Cascade use (ES)	N/A	N/A		N/A	N/A	
					ES		Trade / import Mycoplasma spp. vaccines (ES)	Trade / import Mycoplasma spp. vaccines (ES)						
		Not specified			ES, GR, IT, SE, HR, UK(NI)		Development of vaccines for haemoparasites: Babesia, Theileria Anaplasma (ES)	Development of vaccines for haemoparasites: Babesia, Theileria Anaplasma (ES)						
Antibacterials for systemic use	Tetracyclines	Oxytetracycline	5 (DE)	5 (ES), 4 (DE)	DE	UnmetNat (ES)<br>DE			BG, FR, GR, SK, HU, NL, RO, IE, IT, ES, CY, FI, CZ, LT, DK, DE, PT, AT, HR, PL, SI, LV, SE, EE, IS, BE, LU,	BG, FR, SK, NL, RO, IE, IT, ES, FI, CZ, LT, DK, PT, DE, HR, GR, PL, SI, LV, SE, IS, BE, LU, HU, CY, AT	UnmetNat	BG, FR, IT, ES, PT, RO, EE, GR, CY, LT, LV, HU, PL	BG, FR, PT, RO, ES, GR, CY, LT, IT, HU, PL, LV	UnmetNat
		Doxycycline	4 (GR*, DE*)		UnmetEU (GR*)<br>Form. (DE*)	UnmetEU (DE*, GR*)	Use of APIs effective against respiratory diseases (GR*)<br>There are products licensed for drinking solutions, which is not suitable for ruminants, there are no injection solutions available		/	/	UnmetEU	/	/	UnmetEU
		Chlortetracycline												
		Tetracycline												
		General lack (Not specified)												
	Natural, narrow-spectrum penicillins	Penicillin	4 (DE*)	4 (DE*), 5 (ES)	UnmetNat (DE*)	ES, Dist. (DE*)			N/A	N/A		N/A	N/A	
		Procaine benzylpenicillin*	5 (DE*)		DE*									
	Macrolides	Erythromycin	5 (ES)	4 (DE), 5 (ES)	ES	UnmetEU (ES, DE*)			RO, ES	ES		/	/	UnmetEU
		Tilmicosin	5 (DE*)	4 (DE*)	DE*	UnmetEU (DE*)			UK(NI), RO, SI, AT, BG, LV, ES, LT, PL, HU, IT, BE, CY, PT, SK, CZ, IE, GR, EE, DE, NL, FR, HR, LU	RO, AT, ES, IT, PT, IE, GR, DE, NL, FR, HU, BE, UK(NI), LU		/	/	UnmetEU
		Spiramycin			UnmetEU				/	/	UnmetEU	/	/	UnmetEU
		Tylosin	5 (DE*)		DE*									
		Tulathromycin	5 (DE*), 2 (GR*)	4 (DE*), 2 (GR*)	Reg (GR*)<br>ES, DE*	UnmetEU (ES, DE*, GR*)	Use of other APIs in lactating animals (GR)<br>Off-label use Off-label use carries risk of penalties (ES)	Cascade use (GR)<br>Cascade use Off-label use carries risk of penalties (ES)	AT, BE, BG, HR, CY, CZ, DK, EE, FI, FR, DE, GR, HU, ,IS, IE, IT, LV, LI, LT, LU, MT, NL, NO, PL, PT, RO, SK, SI, ES, SE, UK(NI)	BG, LV, DE, PL, NL, RO, LT, HU, FR, EE, BE, AT, PT, CY, CZ, HR, DK, FI, GR, IE, IT, LU, SK, SE, ES, UK(NI), SI, MT, NO		/	/	UnmetEU
		Gamithromycin*	3 (GR*)	3 (GR*), 4 (DE*)	Reg (GR*)	UnmetEU (GR*, DE*)	Use of other APIs in lactating animals (GR*)	Cascade use (GR*)	ES, FR, UK(NI), DK, SE, IE, AT, BE, BG, HR, CY, CZ, EE, FI, DE, GR, HU, IS, IT, LV, LI, LT, LU, MT, RO, NL, NO, PL, PT, SK, SI	BG, FR, LU, DE, PL, ES		/	/	UnmetEU
		General lack (Not specified)												
	Aminopenicillins, without beta-lactamase inhibitors	Ampicillin	2 (NL*), 4 (DE*)		UnmetNat (DE*, GR*)		Cascade use (NL*)	Cascade use (NL*)	BG, IT, PL, NL, AT, RO, ET, GR, FR, ES, DE	BG, IT, PL, NL, AT, FR, RO, GR	Lack of availability	BG, RO, FR,GR, DE	BG, FR, RO, GR,	Lack of availability
		Amoxicillin	2 (NL*), 5 (DE)	2 (NL*), 4 (DE), 5 (ES)	DE	ES, UnmetNat (DE)	Cascade use (NL*)	Cascade use (NL*)						
		General lack (Not specified)	2 (NL*)		UnmetNat (NL*)		Cascade use (NL*)	Cascade use (NL*)	BG, IT, PL, NL, AT, RO, ET, GR, FR, ES, DE	BG, IT, PL, NL, AT, FR, RO, GR	Lack of availability	BG, RO, FR,GR, DE	BG, FR, RO, GR,	Lack of availability
	Aminoglycosides (except spectinomycin)	Gentamicin	4 (DE*)		UnmetEU	UnmetEU (DE*)			LV	/	Unmet Nat	LV	/	UnmetNat
		Neomycin	4 (GR*, DE*)		UnmetNat (GR*, DE*) Form. (DE*)	UnmetNat (GR*)<br>UnmetNat (DE*)	Off-label use of combinations which<br>Available only as intramammary in Germany		/	/	UnmetEU	RO	/	UnmetNat
		Dihydrostreptomycin												
		Streptomycin				UnmetEU			BG, GR, SK	SK		/	/	UnmetEU
		Apramycin*	4 (DE)		UnmetEU (DE*)				/	/	UnmetEU	/	/	UnmetEU
		Paromomycin	3 (DE*)		UnmetNat (DE*)				/	/	UnmetEU	/	/	UnmetEU
	Cephalosporins	Ceftiofur	2 (NL*)		UnmetEU				/	/	UnmetEU	/	/	UnmetEU
		General lack (Not specified)	2 (NL*)		UnmetEU (NL*)		Cascade (NL)				UnmetEU			UnmetEU
	Quinolones: fluoroquinolones and other quinolones	Norfloxacin	2 (NL*)		UnmetEU (NL*)		Cascade (NL)		/	/	UnmetEU	/	/	UnmetEU
		Enrofloxacin	2 (NL*)		UnmetNat (NL*)		Cascade (NL)		BG, DE, FR, AT, NL, BE, SI, PT, ES, RO, HU, IE, LU, IT, CZ, SK, CY, FI, NO, SE, DK, IS, GR, PL, LV, EE, LT, UK(NI), HR, MT	BG, DE, FR, NL, BE, SI, PT, ES, RO, HU, IE, LU, IT, CZ, SK, CY, FI, NO, SE, DK, IS, GR, LV, LT, AT, HR, PL, UK(NI)	Lack of availability	BG, DE, FR, AT, NL, BE, SI, PT, ES, RO, HU, IE, LU, IT, CZ, SK, CY, FI, NO, SE, DK, IS, GR, PL, LV, EE, LT, UK(NI), HR, MT	BG, DE, FR, NL, BE, SI, PT, ES, RO, HU, IE, LU, IT, CZ, SK, CY, FI, NO, SE, DK, IS, GR, LV, LT, AT, HR, PL, UK(NI)	Lack of availability

		Danofloxacin*	4 (DE*), 2 (NL*)		UnmetEU (DE*, NL*)	UnmetEU (NL*)	Cascade (NL)		/	/	UnmetEU	/	/	UnmetEU
		Marbofloxacin*	2 (NL*), 4 (DE*)		UnmetEU (DE*, NL*)		Cascade (NL)		/	/	UnmetEU	/	/	UnmetEU
		General lack (Not specified)	2 (NL*)		NL* LoA	NL* UnmetNat	Cascade (NL)		N/A	N/A	Lack of availability	N/A	N/A	Lack of availability
	Lincosamides	Lincomycin	4 (DE*)		UnmetNat (DE*)	UnmetEU (DE*)			BG, ES, PT, HU, IT, GR, EE, CZ, SK, ES, RO, BE, FR, LU, LT, LV, PL	BG, HU, CZ, GR, SK, ES, RO, BE, LU, IT, LV, LT, FR	UnmetNat	BG, GR, HU, EE, RO, FR, LV, SK, LT, CZ, PT, IT, PL	BG, HU, GR, RO, LV, LT, IT, FR	UnmetNat
	Sulfonamides, dihydrofolate reductase inhibitors and combinations	Trimethoprim; Sulfamethoxazole	2 (NL*)		UnmetEU (NL*)		Cascade (NL)		RO, LT	/	UnmetNat	RO, LT	/	UnmetNat
		Trimethoprim; Sulfadoxine	4 (SE, GR*), 5 (DE*)		DE*, Reg (GR*)	Reg (GR*)	Cascade use (SE, GR*)							
		General lack (Not specified)			UnmetNat (SE)		Authorization (SE)		N/A	N/A	UnmetNat	N/A	N/A	UnmetNat
	Polymyxins	Colistin												
	Amphenicols	Florfenicol	3 (GR*), 4 (DE*)		Reg (GR*)	UnmetEU (DE*, GR*)	Use of other APIs in lactating animals (GR*)	Cascade use (GR*)	CZ, ES, BE, HR, DK, EE, FI, FR, HU, GR, IT, LV, NL, PL, PT, SK, UK(NI), CY, BG, AT, LT, SI, DE, IE, RO, SE, LU	ES, HR, DK, EE, FR, GR, IT, LV, CY, BG, AT, LT, DE, IE, PL, RO, NL, PT, UK(NI), BE, LU		/	/	UnmetEU
	Aminoglycosides: spectinomycin only	Spectinomycin												
	Aminopenicillins, in combination with beta lactamase inhibitors	Amoxicillin; Clavulanic acid	2 (GR*), 4 (DE*)		UnmetEU (DE*, GR*)		Use of amoxicillin (GR*)		/	/	UnmetEU	/	/	UnmetEU
	Combination of antibacterials	Dihydrostreptomycin; Penicillin												
		Penicillin; Streptomycin			DE*									
	Combination of antibacterials and corticosteroid	Dexamethasone; Dihydrostreptomycin; Penicillin			UnmetEU				/	/	UnmetEU	FR, LV	FR, LV	
	Macrolides, combination with other substances	Ketoprofen; Tulathromycin			UnmetEU				/	/	UnmetEU	/	/	UnmetEU
	Non-specified antibiotics classes	AMEG Group D antibiotics (non-specified)			ES									
		Antibiotics for anaplasmosis (non-specified)			ES				N/A	N/A		N/A	N/A	
		Antibiotics for diarrhea (non-specified)			Reg (ES)		Enrofloxacin off-label use not possible due to							
		Antibiotics for pneumonia (non-specified)			ES									
		Antibiotics for colibacillosis (non-specified)			ES									
		Antibiotics for Mycoplasma pneumonia (non-specified)			ES									
		Antibiotics for mastitis (non-specified)			ES									
		General lack (not specified)	2 (NL)		Dist (ES, DE, FR, NL)		Cascade (NL)		N/A	N/A	Lack of availability	N/A	N/A	Lack of availability
Antibacterials for local use	Antibacterials for intra-mammary use	Clinical mastitis injectors (non-specified)	5 (NL)	1 (NL)	Reg (ES); UnmetNat (GR); IT, NL	UnmetEU (ES, GR, IT, NL)					UnmetNat			UnmetNat
		Subclinical mastitis injectors (non-specified)			Reg. (ES)				BG, IT, FR, LT, ES, CY, GR, DE, NL, PO, PT	BG, ES,GR, FR, IT, DE, NL		BG, IT, LT, CY, GR, FR	BG, GR, FR	
		Mastitis injectors for dry period			ES									
		Intramammary antibacterials in general	2 (GR)		UnmetNat (GR)	UnmetEU (GR)	Trade / import / Off-label	Cascade use			UnmetNat			UnmetNat
	Antibacterials for ocular use	Cloxacillin	4 (DE)		UnmetNat (DE*)	UnmetEU (DE*)			BG, RO, NL, IE, LV, DE	RO, NL, IE, DE	UnmetNat	RO	RO	UnmetEU
		General lack (Not specified)				UnmetEU			BG, CY, DE, IE, LV, LT, NL	DE, IE, LT, NL		/	/	UnmetEU
	Antibacterials for topical use	General lack (Not specified)			ES									
	Antibacterials for genito-urinary use	General lack (Not specified)												
Nonsteroidal anti-inflammatory drugs	Propionic acid	Ketoprofen	2 (GR*), 4 (DE*)		UnmetEU (GR*)		Cascade use (GR*, DE*)		/	/	UnmetEU	/	/	UnmetEU
	Oxicams	Meloxicam	1 (GR*, DE*)		UnmetEU (DE*, GR*)		Cascade use (GR*)		/	/	UnmetEU	/	/	UnmetEU
	Fenamates	Flunixin	2 (GR*), 4 (DE*)		UnmetEU (DE*, GR*)		Cascade use (GR*)		/	/	UnmetEU	/	/	UnmetEU
	NSAIDs in general	General lack (Not specified)	4 (FR, SE), 1 (NL)		UnmetEU (ES, DE, FR, NL, SE, UK(NI))		Cascade use (FR, Withdrawal period Not possible in organic farming in France		/	/	UnmetEU	/	/	UnmetEU
Corticosteroids	Glucocorticoids	Dexamethasone	2 (NL, GR*), 5 (DE*)		UnmetEU (ES, GR*)	UnmetNat (GR*) DE*, ES, NL	Cascade use (NL, GR*)		LV, GR	GR	UnmetNat	BG, SI, LV, IT, AT, HR, CZ, DK, EE, DE, GR,	BG, AT, EE, IS, NL, BE, LV, FR, LU, RO, IT, DE,	
		Cortisone	2 (NL)		UnmetEU (NL, DE)		Cascade use (NL)		/	/	UnmetEU	/	/	UnmetEU
		Prednisolone*	2 (NL), 4 (DE*)		UnmetEU (NL)		Cascade use (NL,		/	/	UnmetEU	/	/	UnmetEU
	Glucocorticoids in general	General lack (Not specified)	2 (NL)		UnmetNat (ES, NL)	ES, NL	Cascade use (NL)				UnmetNat			
Other analgesics and antipyretics	Pyrazolones	Metamizole			UnmetEU				/	/	UnmetEU	/	/	UnmetEU
Analgesics and antipyretics	Analgesics and antipyretics in general	General lack (Not specified)			UnmetEU (ES, DE, FR)				/	/	UnmetEU	/	/	UnmetEU

Topical and ocular anti-inflammatory medicines	Topical and ocular anti-inflammatory medicines in general	General lack (Not specified)			UnmetNat (ES)				BG	/	UnmetNat	BG	/	UnmetNat
Intramammary anti-inflammatory medicines	Intramammary anti-inflammatory medicines in general	General lack (Not specified)			UnmetEU				/	/	UnmetEU	/	/	UnmetEU
Anti-inflammatory medicines	Anti-inflammatory medicines in general	General lack (Not specified)			UnmetNat (ES)	ES			/	/	UnmetNat	/	/	UnmetEU
	Benzimidazoles	Albendazole	1 (DK), 5 (DE*)	2 (DK), 4 (DE)	Dist. (DK)	UnmetNat (DK, DE*)	Cascade use (DK)<br>Dosages for cascade use in goats should be available.<br>Cascade use (DE)<br>Cascade use in Germany is possible with presented proof of resistance to eprinomectin		BG, ES, GR, RO, HU, SK, DE, LV, EE, CY, FR, PT, IT, SI, AT, HR, LT, IE, CZ, PL, DK, SE, NO	BG, SK, ES, DE, RO, GR, PT, IT, DK, SE, NO	UnmetNat	BG, ES, RO, GR, LT, PT, FR, CY	BG, RO, GR, PT	UnmetNat
		Netobimin	1 (ES)		UnmetEU (ES)		Oral suspension for sheep and goats is available on national level (ES)		IT	/	UnmetNat	/	/	UnmetEU
		Fenbendazole*	2 (HR*), 5 (DE*)	2 (HR*), 4 (DE*)	UnmetNat (HR*)	UnmetNat (HR*, DE*)	Registration / interventional Trade / import.<br>Rotation of medicine groups. (HR*)<br>Resistance present in alternatives.<br>Cascade use (DE)<br>Cascade use in Germany is possible with presented proof of resistance to eprinomectin		IE, SE, LT, NO, GR, RO, HU, DE, SK, ES, FR, PT, IT, AT, LU, BE, NL, CY, DK, CZ	SE, DE, RO, ES, FR, PT, IT, GR, AT, BE, NL, CY, LU, DK, NO, CZ	UnmetNat	NO, SK, ES, FR, PT, IT, GR, RO, CZ	ES, FR, PT, IT, GR, NO, CZ	UnmetNat
		Triclabendazole*	1 (GR*), 5 (DE*)	1 (GR*), 4 (DE*)	UnmetNat (GR*)	UnmetEU (GR*)	Use of albendazole. (GR*)<br>Resistance present in alternatives.<br>Cascade use<br>Cascade only possible in non-lactating goats		See results in row 233, repetition of active substance	See results in row 233, repetition of active substance		See results in row 233, repetition of active substance	See results in row 233, repetition of active substance	
		Mebendazole*	3 (HR*)		UnmetNat (HR*)	UnmetEU (HR*)	Registration / interventional Trade / import.<br>Use of combination products containing mebendazole.<br>Rotation of medicine groups. (HR*)<br>Resistance present in alternatives.	Registration / interventional Trade / import.<br>Cascade use of combination products containing mebendazole<br>Rotation of medicine groups. (HR*)<br>Resistance present in alternatives.	IE, BE, AT, DE, NL, IS, IT, PT, ES, FR	IE, BE, AT, DE, NL, IT, PT, ES, FR	UnmetNat	/	/	UnmetEU
		General lack (Not specified)												
		Closantel	2 (HR*)		UnmetNat (HR*)	UnmetEU (HR*)	Registration / interventional Trade / import.<br>Rotation of medicine groups. (HR*)<br>Resistance present in alternatives.		/	/	UnmetEU	/	/	UnmetEU
							Registration / interventional Trade / import.<br>Rotation of medicine groups. (HR*)							

Anthelmintics	Salicylanilides	Oxyclozanide*	1 (HR*), 5 (DE*)	1 (HR*), 4 (DE)	HR*	UnmetNat (HR* DE*)	Resistance present in alternatives.<br>Cascade use (DE)<br>Cascade use in Germany is possible with presented proof of resistance to eprinomectin		BG, UK(NI), PL, SE, LU, NO, RO, SI, DE, HU, BE, DK, NL, PT, IE, IT, AT, HR, FR, GR, ES	UK(NI), PL, RO, DE, HU, BE, NL, IE, IT, AT, HR, FR, BG, GR	UnmetNat	BG, RO	RO	UnmetNat
		Rafoxanide*	5 (GR*). 3 (DE*)	4 (GR*), 3 (DE*)	UnmetNat (DE*)	UnmetEU (GR*)	Cascade use (DE)	Use of albendazole. (GR*)<br>Resistance present in alternatives.<br>Available only in Greece	LT, IE, GR	GR	UnmetNat	/	/	UnmetEU
	Imidazothiazoles / Tetrahydropyrimidines	Levamisole	3 (HR*), 5 (DE)	3 (HR*), 1 (DE)	DE, UnmetNat (HR*)	UnmetEU (DE, HR*)	Registration / interventional Trade / import.<br>Rotation of medicine groups. (HR*)<br>Resistance present in alternatives.		BG, ES, FR, IE, DE, LT, NL, RO, LV, GR, PL, IT	BG, ES, FR, NL, IE, DE, RO, GR	UnmetNat	BG	/	UnmetNat
		Pyrantel*	1 (HR*)		HR* UnmetEU	HR* UnmetEU	Registration / interventional Trade / import.<br>Rotation of medicine groups. (HR*)<br>Resistance present in alternatives.		/	/	UnmetEU	/	/	UnmetEU
	Trematocides (non-specified)	For *Dicrocoelium* spp.			ES	ES, IT								
		For *Fasciola* spp.			ES	ES, IT								
		General lack (Not specified)			FR	FR								
	Amino-acetonitrile derivatives (AADs)	Monepantel	5 (DE*)	4 (DE*)		UnmetEU	Cascade use (DE)<br>Cascade use in Germany is possible with presented proof of resistance to eprinomectin		AT, BE, BG, HR, CY, CZ, DK, EE, FI, FR, DE, GR, HU, IS, IE, IT, LV, LI, LT, LU, MT, NL, NO, PL, PT, RO, SK, SI, ES, SE, UK(NI)	AT, BE, FR, DE, IE, LU, NL, UK(NI)	UnmetNat	/	/	UnmetEU
	Quinoline derivatives and related substances	Praziquantel	5 (DE)	4 (DE)	DE	UnmetEU (DE)			DE, PT, GR, FR, IT, IE, RO, LT, CZ	DE, PT, GR, FR, IT, RO	UnmetNat	/	/	UnmetEU
	Combination of benzimidazole and related substances	Levamisole; Triclabendazole				UnmetEU			AT, FR, BE, DE, IE, LU, NL, PT, SE, GB, RO, HR, HU, ES, GR, IT	AT, BE, DE, IE, LU, NL, SE, UK(NI)	UnmetNat (Triclabendazole)	/	/	UnmetEU (Triclabendazole)
	Anthelmintics in general	General lack (Not specified)	2 (BE), 5 (NL), 1 (SE)	2 (BE), 1 (NL, SE)	Efs. (NL, BE)		Prudent antiparasitic use.<br>Rotation of antiparasitic classes.<br>Registration of single API products.<br>Cascade use<br>Dosages for cascade use in goats should be available.<br>Sole reliance on combination products disable prudent anthelmintic use (NL, BE, SE)							
					ES, DE		Vaccine development (BE)							
					Dist. (SE)		Improve marketing frequency (SE)							
							Cascade use (DE)	Cascade use (GR*) Cascade use (DE*)						

		Doramectin	5 (DE*)	4 (DE*), 3 (GR*)	UnmetNat (DE*)	UnmetEU (DE*, GR*)	Cascade use in Germany is possible with presented proof of resistance to eprinomectin	Cascade use in Germany is possible with presented proof of resistance to eprinomectin	BG, HU, NL, SI, LV, IE, AT, CY, CZ, EE, ES, GR, FR, HR, NO, PT, SK, PL, RO, LT, IT, DK, DE, BE	HU, NL, IE, BG, ES, GR, FR, NO, SK, PL, RO, IT	UnmetNat	/	/	UnmetEU
Endectocides	Macrocyclic lactones	Moxidectin	1 (DK), 5 (DE*)	2 (DK), 4 (GR*, DE*)	DIst. (DK)<br>UnmetNat (DE*)	UnmetEU (DK, DE*, GR*)	Cascade use (DK)<br>Dosages for cascade use in goats should be available.	Cascade use (GR*)<br>Cascade use in Germany is possible with presented proof of resistance to eprinomectin	BE, DK, DE, GR, IE, IT, LU, NL, ES, UK(NI), AT, PT, FR, SE, PL, BG, CY, NO, HR	BE, DE, GR, IE, IT, LU, NL, ES, UK(NI), AT, SE	UnmetNat	/	/	UnmetEU
		Eprinomectin	5 (DE*)	1 (GR*), 5 (DE*)	DE*	DE*, GR*		Cascade use (GR*)						
		Ivermectin		4 (DE*)	ES	ES, GR*<br>UnmetNat (DE*)		Cascade use in Germany is possible with presented proof of resistance to eprinomectin	BG, IE, GR, ES, UK(NI), LV, FR, EE, LT, PL, CZ, RO, HR, SK, HU, NL, PT, BE, AT, DE, IT, LU, DK, CY, SI, NO, SE, IS	PL, HR, SK, HU, IE, RO, SI, ES, PT, LU, BE, AT, CY, FR, SE, NO, DK, IS, BG, NL, CZ, GR	UnmetNat	BG, EE, HR, RO, LT, SE, NO, IS, LV, ES, IT, GR, SK	RO, SE, NO, IS, ES, GR	UnmetNat
		Abamectin*	2 (DE*)		UnmetEU (DE*)		Only in mixture in UK		BG, HR, RO, IE, UK(NI)	UK(NI)	UnmetNat	/	/	UnmetEU
		General lack (Not specified)												
	Endectocides in general	General lack (Not specified)												
	Other endectocides	Clorsulon*	3 (GR*)		UnmetEU (GR*)		Use of albendazole.<br>Resistance present in alternatives		/	/	UnmetEU	/	/	UnmetEU
	Coccidiostats (triazines)	Toltrazuril	1 (DE*), 5 (NL*)	3 (DE*), 2 (GR*, NL*)	DE*	UnmetEU (DE*, GR*, NL*)		Cascade use (NL, GR*)<br>Withdrawal period in relclassification for use in goats too long in Germany	IS, HU, NO, NL, LV, PL, SI, SK, RO, SE, PT, UK(NI), IE, BE, AT, CY, BG, CZ, DK, DE, EE, ES, FI, FR, LT, IT, HR, LU, GR	IS, HU, NO, NL, LV, PL, SI, SK, RO, SE, PT, UK(NI), IE, BE, AT, CY, BG, CZ, DK, DE, EE, ES, FI, FR, LT, IT, HR, LU, GR	UnmetNat	/	/	UnmetEU
		Diclazuril	5 (DE*)	4 (DE*), 2 (GR*)	UnmetEU (DE*)	UnmetEU (DE*, GR*)		Cascade use (GR*)	ES, PT, CZ, IT, AT, PL, BE, LT, EE, UK(NI), LV, FR, IE, DE, NL, RO, SI, BG, HR, CY, HU, GR, LU, DK	CY, ES, HU, LU, PT, SK, FR, AT, BE, CZ, IE, NL, GR, IT, DE	UnmetNat	/	/	UnmetEU
	Coccidiostats (other)	Halofuginone			UnmetEU				/	/	UnmetEU	/	/	UnmetEU
	Other agents against coccidiosis	Decoquinate	3 (DE*)		Reg. (ES)	UnmetEU (ES, DE*)	Trade from Spain (DE*)		UK (NI), ES, IT, PT, IE, FR	UK (NI), ES, IT, PT, IE, FR	UnmetNat	/	/	UnmetEU
Antiprotozoal medicines and coccidiostats	Coccidiostats in general	General lack (Not specified)			ES	UnmetEU (ES)	Diclazuril and toltrazuril available for sheep (ES)							UnmetEU
		Aminosidine sulphate*			UnmetEU				/	/	UnmetEU	/	/	UnmetEU
		Treatment for *Cryptosporidium* spp. (non-specified)			Reg. (ES)		Product registered only for: pre-ruminant lambs and pre-ruminant kids							
	Antiprotozoal medicines	Treatment for *Babesia* spp. (non-specified)	1 (ES*)		Efs. (ES*)		Off-label use of imidocarb dipropionate		N/A	N/A		N/A	N/A	
		Treatment for *Theileria* spp. (non-specified)	1 (ES*)		Efs. (ES*)				N/A	N/A		N/A	N/A	
		Treatment for *Anaplasma* spp. (non-specified)	1 (ES*)		Efs. (ES*)				BG, FR, RO, DE, IE, CY, GR, ES, SK, HR, CZ, NL, HU, IT, FI, LT, DK, EE, PT, AT, SI, PL, LV, UK(NI), BE, LU, SE, IS, NO	BG, FR, RO, DE, IE, HR, CZ, NL, HU, SK, IT, ES, FI, LT, DK, PT, SI, CY, GR, PL, LV, AT, BE, LU, SE, IS, NO		BG, FR, IT, ES, PT, RO, HR, SI, LT, EE, CZ, GR, CY, LV, HU, NO, PL	BG, FR, PT, HR, SI, RO, ES, GR, CY, HU, LT, IT, NO, LV, PL	
							Registration / interventional Trade / import.							

Ectoparasiticides	Pyrethrin and pyrethroids	Cypermethrin	2 (HR*)		UnmetNat (HR*)		Rotation of medicine groups. (HR*) Resistance present in alternatives.		BG, ES, PT, RO, IE, NO, FR, UK(NI), NL, CY, GR	ES, RO, NO, IE	UnmetNat	BG, PT, ES, CY, GR	/	UnmetNat
		Deltamethrin	5 (DE)	4 (DE)	DE	UnmetNat (DE)			BG, FR, ES, RO, IT, PT, IE, GR, BE, CY, FI, SI, SE, NL, LU, AT, DE, DK, NO, PL, EE, HU, IS, LV, LT, SK, UK(NI), CZ	FR, ES, IT, PT, IE, GR, BE, FI, SI, SE, NL, LU, AT, DE, DK, NO, EE, HU, LV, LT, PL, RO, SK, UK(NI), BG, CZ		BG	/	UnmetNat
		Not specified				LoA			N/A	N/A		N/A	N/A	Lack of availability
	Organophosphates	Phoxim												
		Diazinon*	1 (NL*)		UnmetNat (NL*)		Cascade use (NL)		/	/	UnmetEU	/	/	UnmetEU
	Isoxazolines	Fluralaner*	4 (DE*)		UnmetEU	UnmetEU (DE*)	Cascade use (DE*)		/	/	UnmetEU	/	/	UnmetEU
	Repellents	Not specified	1 (DE*)		UnmetEU (DE*)		Authorization (DE*) Only insecticides available, not suitable for prevention of infectious diseases transmission				UnmetEU			UnmetEU
	Ectoparasiticides in general	Sheep scab medicine (Phoxim)	5 (DE)	4 (DE*)	DE	DE*								
		General lack (Not specified)			DE									
Antiparasitic products, including ectoparasiticides in general	Not specified	General lack (Not specified)	4 (FR)		Reg. + Efs. + Dist. (FR, HR)		Registration / Trade / import.		N/A	N/A		N/A	N/A	
							Withdrawal period adjustment		N/A	N/A		N/A	N/A	
					Reg. (ES)				N/A	N/A		N/A	N/A	
					DE	FR			N/A	N/A		N/A	N/A	
	Topical antiparasitics	General lack (Not specified)							N/A	N/A		N/A	N/A	
Drugs used in diabetes	Insulins and analogues	Insulin	1 (DE*)		UnmetEU (DE*)		Can not be solved by the cascade (DE*)		/	/		/	/	
Pituitary and hypothalamic hormones and analogues	Posterior pituitary lobe hormones	Oxytocin	2 (SE*), 5 (DE*)		UnmetNat (SE*)		Authorization of sheep and goat products Better marketing frequency for cascade use (SE*)		BG, NL, FR, PL, HU, ES, PT, LT, GR, CY, IE, AT, HR, IT, RO, EE, CZ, SK, UK(NI), NO, DK, IS, DE, LV, SI, BE, FI	FR, ES, PT, GR, RO, CY, EE, HR, HU, UK(NI), NO, IE, DK, IS, DE, LV, SK, LT, SI, BE, NL, PL, FI	UnmetNat	BG, NL, FR, PL, HU, ES, PT, LT, GR, IT, RO, CY, EE, CZ, HR, SK, UK(NI), NO, IE, DK, IS, AT, DE, LV, SI, BE	FR, ES, PT, GR, RO, CY, EE, HR, HU, UK(NI), NO, IE, DK, IS, DE, LV, SK, LT, SI, BE, NL, PL	UnmetNat
	Hypothalamic hormones	Gonadotropin-releasing hormones		5 (ES)	UnmetEU	UnmetEU (ES)			/	/	UnmetEU	/	/	UnmetEU
Uterotonics	Prostaglandins	Cloprostenol	2 (GR*), 4 (DE*)		UnmetEU (FR, DE*, GR*)		Cascade use (GR*, DE*)		BG	/	UnmetNat	BG, GR, NO, SE, FI, DE, PL, PT, NL, RO, FR, IT, ES, UK(NI), IE, AT, SI, SK, BE, LU, HU, HR, CZ, LV, EE, DK, LT	FR	UnmetNat
		Luprostiol*			UnmetEU				/	/	UnmetEU	/	/	UnmetEU
		Prostaglandine F2alpha**	4 (DE*)	5 (DE*)	UnmetEU		Cascade use (DE*)		The name of the below medication is Prostaglandine F2A, however the active substance is listed as prostaglandin A1	/	UnmetEU	/	/	UnmetEU
		Prostaglandins in general (not specified)	5 (ES)		ES	UnmetEU (ES)			GR	/	UnmetNat	/	/	UnmetNat
Sex hormones and modulators of the genital system	Progestogens	Flugestone			UnmetEU				BG, FR, RO, CY, PT, ES, UK(NI), GR, IE, SK, IT	FR, RO, PT, ES, UK(NI), GR, IE, SK, IT	UnmetNat	GR, RO, IT, FR	RO, IT	UnmetNat
		Progesterone*	4 (GR*), 3 (SE*)		UnmetNat (GR*, SE*)	DE*, UnmetEU (GR*, SE*)	Cascade use (GR*, Authorization (SE*)		RO	RO	UnmetNat	RO	RO	UnmetNat
		Progesterone (sponges)*	1 (DE*)	1 (GR*, DE*)		UnmetEU(DE*, GR*)	Trade / import (DE*)	Cascade use (GR*), Trade / import (DE*)	ES, CY, FR, GR, IT, PT, UK(NI), NL	ES, CY, GR, IT, UK(NI), NL		/	/	UnmetEU
	Gonadotropins	Pregnant Mare Serum Gonadotropin*	5 (DE*), 3 (SE*)	4 (DE*), 3 (SE*)	UnmetNat (SE*) DE*	UnmetNat (DE*, SE*)	Authorization Research and development of synthetic alternatives (SE*)		GR, FR, IT, LU, NL, CY, PT, RO, ES	FR, IT, LU, NL, CY, GR, PT	UnmetNat	GR, FR, IT, LU, NL, CY, RO	FR, IT, LU, NL, CY, GR	UnmetNat
	Sex hormones in general	Not specified							N/A	N/A		N/A	N/A	
Other gynecologicals	Sympathomimetics, labour repressants	Clenbuterol	1 (DE)		UnmetEU (DE)				/	/	UnmetEU	/	/	UnmetEU
Hormonal preparations	Hormonal preparations in general	Not specified	3 (GR)		GR		Cascade use (GR)		N/A	N/A		N/A	N/A	
	Local anesthetics	Lidocaine	4 (DE*), 3 (GR*)		UnmetEU (DE*, GR*)		Cascade use (GR*,		/	/	UnmetEU	/	/	UnmetEU
		Procaine	5 (DE*)	4 (DE*)		UnmetEU		Cascade use (DE*)	IT, ES, DE, BE, LU, SI, LV, LT, SK, DK, SE, NL, PT, FI, AT, CZ, FR, IS, NO, EE, RO, IE, BG, HU, PL, HR, UK(NI), GR, CY	IT, BE, LU, SI, LV, LT, SK, DK, SE, NL, PT, FI, AT, DE, FR, IS, NO, EE, ES, CZ, HU, PL, RO, UK(NI), IE, GR		DE, RO, AT	/	UnmetNat
		Local anesthetics in general (not specified)				UnmetNat								
		Ketamine	5 (DE*)											
							Cascade use (DE*)							

Anesthetics	General anesthetics and combinations	Isoflurane*	4 (DE*)		UnmetEU DE*		Cannot be used in animals for production of milk for human consumption (DE*)		/	/	UnmetEU	/	/	UnmetEU
		Propofol*	3 (DE*)		UnmetEU DE*				/	/	UnmetEU	/	/	UnmetEU
		Tiletamine; Zolazepam			UnmetEU				/	/	UnmetEU	/	/	UnmetEU
	Anesthetics in general	Not specified	5 (NL)	2 (NL)	LoA (ES), NL		Cascade use (ES, NL)				Lack of availability			Lack of availability
Hypnotics and sedatives	Alpha-2 adrenergic agonist	Xylazine	4 (GR*)		UnmetNat (GR*)	UnmetEU (DE*, GR*)	Cascade use (GR*, DE*)		SE, LT, LV	SE	UnmetNat	/		UnmetEU
	Tranquilizers	Tranquilizers in general (not specified)			(ES)	(ES)	Cascade use (ES)							
	Melatonin receptor agonists	Melatonin*	2 (DE*)		UnmetEU	UnmetEU (DE*)			BG, FR, ES, PT, IT, IE, RO, GR	/		ES, GR	/	
	Hypnotics and sedatives in general	Not specified				UnmetNat						GR	/	UnmetNat
Antipsychotics	Phenothiazines	Acepromazin*	4 (DE*)		UnmetEU		Can not be solved by the cascade in Germany (DE*)		/	/	UnmetEU	/	/	UnmetEU
Anxiolytics	Benzodiazepines	Diazepam*	1 (DE*)		UnmetEU DE*				/	/	UnmetEU	/	/	UnmetEU
Opioids	Morphinan derivatives	Butorphanol*	4 (DE*)		UnmetEU DE*		Cascade use (DE*)		/	/	UnmetEU	/	/	UnmetEU
	Opioids in general	Not specified			UnmetEU (DE)						UnmetEU			UnmetEU
Products for animal euthanasia	Barbiturates	Pentobarbital*	5 (DE*)		UnmetEU				DE, LV, BE, BG, CZ, DK, EE, FI, HU, IE, IT, LT, MT, PT, RO, SI, ES, SE, UK(NI), PL, NL, CY, GR, LU, NO, SK, AT, HR, FR, IS	DE	Lack of availability	DE, LV, BE, BG, CZ, DK, EE, FI, HU, IE, IT, LT, MT, PT, RO, SI, ES, SE, UK(NI), PL, NL, CY, GR, LU, NO, SK, AT, HR, FR, IS	DE	Lack of availability
Vitamins	Vitamins, plain	Vitamin B12	2 (GR*), 5 (DK*)		UnmetEU (GR*, DK*)		Cascade use (GR*, DK*)<br>Available only in mixtures (multivitamins)		IE, LV, IT, LT, PT, HU	LV, PT	UnmetNat	IT, LT, PT	PT	UnmetNat
		Vitamin B1	1 (DE*), 2 (GR*), 5 (DK*)		UnmetEU (DE*, GR*, DK*)		Dosages for cascade use in goats should be available.		/	/	UnmetEU	/	/	UnmetEU
	Vitamin B-complex including combinations	Vitamin B complex	5 (DK*)		UnmetNat (DK*)<br>Form (DE*)		Cascade use (DK*)<br>Dosages for cascade use in goats should be available.<br>Plain vitamins needed also (DE*)		IE, LV, IT, LT, PT, HU, ES	LV, PT	UnmetNat	IT, LT, PT, ES	PT	UnmetNat
	Multivitamins, plain	Not specified			ES									
	Vitamins, Intramammary	Not specified			UnmetEU				/	/	UnmetEU	/	/	UnmetEU
	Vitamins in general	Not specified			ES									
	Injectable vitamins	Not specified			Reg. (ES)	ES								
Minerals	Minerals, plain	Selenium	5 (DE*)		DE*									
		Copper*	1 (DE*)		UnmetEU DE*				/	/	UnmetEU	/	/	UnmetEU
		Calcium												
		Calcium (subcutaneous)			SE									
	Minerals in combination with other compounds	Calcium gluconate												
		Calcium borogluconate	5 (DE*)		DE*									
		Calcium borogluconate; Magnesium Hypophosphate*	5 (DE*)		DE*									
		Copper Glycinate*	3 (DE*)		UnmetEU				/	/	UnmetEU	/	/	UnmetEU
		Copper EDTA*	3 (DE*)		UnmetEU DE*				/	/	UnmetEU	/	/	UnmetEU
		Magnesium sulfate*	5 (DE*)		DE*									
	Minerals in general	Not specified												
Vitamin and mineral combinations	Vitamins with minerals	Butaphosphan; Vitamin B12			UnmetEU				IE, LV, IT, LT, PT, HU	LV, PT	UnmetNat	PT	PT	UnmetNat
		Selenium; Vitamin E												
		General lack (Not specified)	2 (DE*)		DE*		Use of feed additives (DE*)							
	Multivitamins, other combinations	Levocarnitine; Lipoic acid; Vitamin B6; Vitamin B12; Acetylmethionine; Arginine; Ornithine; Citrulline; Lysine;Glycine; Aspartate, Glutamate; Fructose; Sorbitol												

Antiseptics and disinfectants	Biguanides and amidines	Chlorhexidine*	5 (DE*)	4 (DE*)	DE*									
	Antiseptics and disinfectants in general	Not specified												
Biologicals	Biologicals in general	Not specified												
Blood substitutes and perfusion solutions	Intravenous solutions	Dextrose 5%*	4 (DE*)		DE*									
		Lactated ringers' solution**	5 (DE*)		DE*									
		NaCl - 0,9%**	5 (DE*)		DE*									
		Glucose*	5 (DE*)		DE*									
		Propylene glycol**	4 (DE*)				Use of feed additives (DE*)		FR, NL, IE		UnmetEU	IE		UnmetEU
		Intravenous solutions in general (not specified)							N/A	N/A		N/A	N/A	
Respiratory system products	Respiratory stimulants	Doxapram hydrochloride*	4 (HR*)		UnmetEU (HR)		Registration / interventional Trade		DE	/	UnmetEU	/	/	UnmetEU
	Mucolytics	Bromhexine			UnmetEU		Cascade use (ES)	Cascade use (ES)	/	/	UnmetEU	/	/	UnmetEU
		Mucolytics in general			UnmetEU	UnmetEU (ES)			/	/	UnmetEU	/	/	UnmetEU
Dermatologicals	Products for the treatment of claws and hoofs	Zinc sulphate bath	1 (DE*)		UnmetEU (DE)				/	/	UnmetEU	/	/	UnmetEU
		Copper sulphate bath*	1 (DE*)		UnmetEU	UnmetEU DE*			/	/	UnmetEU	/	/	UnmetEU
		Foot rot bath (not specified)	1 (DE)		DE		Use of alternatives:		N/A	N/A		N/A	N/A	
							Copper concentration too low in existing products (DE)		N/A	N/A		N/A	N/A	
	Antifungals for dermatologic al use	Antifungals for dermatomicosis in general (not specified)	1 (ES)		UnmetEU (ES)		Use of quaternary ammonium or sulfur oil (ES)		/	/	UnmetEU	/	/	UnmetEU
Alimentary tract and metabolism	Drugs for functional gastrointestin al disorders	Propulsives in general (not specified)	3 (NL)		UnmetEU (NL)		Cascade use (NL)		/	/	UnmetEU	/	/	UnmetEU
	Metabolics in general	Not specified				DE			N/A	N/A		N/A	N/A	
Sensory organs	Ophthalmolo gicals	Not specified			FR	UnmetEU (FR)			N/A	N/A		N/A	N/A	UnmetEU
Diagnostic agents	Tuberculosis diagnostics	Tuberculin bovine*	5 (DE*)		DE*				BG, RO, LV, HU, LT, CZ, SK, DE	DE, RO	UnmetNat	BG, RO, LV, HU, LT, CZ, SK, DE	DE, RO	UnmetNat
		Tuberculin avian*	1 (DE*)		UnmetEU DE*				GR, PT, ES, IS, NO, FR, NL, IT	GR, PT, ES, IS, NO, NL		GR, PT, ES, IS, NO, FR, NL, IT	GR, PT, ES, IS, NO, NL	
	X-ray contrast media, non-iodinated	Barium sulfate*	1 (DE*)		UnmetEU DE*				/	/	UnmetEU	/	/	UnmetEU
Miscellaneous	Unspecified sheep and goat medication	Diarrhea medicine			ES				N/A	N/A		N/A	N/A	
		Pneumonia medicine							N/A	N/A		N/A	N/A	
		Enteritis medicine							N/A	N/A		N/A	N/A	
		Not specified	4 (SE)		ES, DE, IT, NL, SE	ES, DE, IT, NL, SE, UK(NI)			N/A	N/A		N/A	N/A	
	Medicines applicable during lactation	Not specified	2 (GR*)		UnmetNat (GR*)		Trade / import / Off-label use (GR)	Cascade use (GR)	N/A	N/A	UnmetNat	N/A	N/A	UnmetNat
					DE				N/A	N/A		N/A	N/A	
	Intramammar y products in general	Not specified	1 (DE), 2 (GR), 5 (NL)	1 (NL, DE), 2 (GR)	UnmetNat (GR)	UnmetNat (NL, GR)	Cascade use (NL, GR)		N/A	N/A	UnmetNat	N/A	N/A	UnmetNat
					DE, IT				N/A	N/A		N/A	N/A	
